# 11-Keto-α-Boswellic Acid, a Novel Triterpenoid from *Boswellia* spp. with Chemotaxonomic Potential and Antitumor Activity against Triple-Negative Breast Cancer Cells

**DOI:** 10.3390/molecules26020366

**Published:** 2021-01-12

**Authors:** Michael Schmiech, Judith Ulrich, Sophia Johanna Lang, Berthold Büchele, Christian Paetz, Alexis St-Gelais, Tatiana Syrovets, Thomas Simmet

**Affiliations:** 1 Institute of Pharmacology of Natural Products and Clinical Pharmacology, Ulm University, 89081 Ulm, Germany; michael.schmiech@uni-ulm.de (M.S.); judith.ulrich@uni-ulm.de (J.U.); sophia.lang@uni-ulm.de (S.J.L.); berthold.buechele@t-online.de (B.B.); 2 Max Planck Institute for Chemical Ecology, 07745 Jena, Germany; cpaetz@ice.mpg.de; 3 Laboratoire PhytoChemia, Chicoutimi, QC G7J 1H4, Canada; a.st-gelais@phytochemia.com

**Keywords:** *Boswellia*, frankincense, boswellic acid, KBA, AKBA, triterpenoids, breast cancer, TNBC

## Abstract

Boswellic acids, and particularly 11-keto-boswellic acids, triterpenoids derived from the genus *Boswellia* (*Burseraceae*), are known for their anti-inflammatory and potential antitumor efficacy. Although boswellic acids generally occur as α-isomers (oleanane type) and β-isomers (ursane type), 11-keto-boswellic acid (KBA) was found only as the β-isomer, β-KBA. Here, the existence and natural occurrence of the respective α-isomer, 11-keto-α-boswellic acid (α-KBA), is demonstrated for the first time. Initially, α-KBA was synthesized and characterized by high-resolution mass spectrometry (HR-MS) and nuclear magnetic resonance (NMR) spectroscopy, and a highly selective, sensitive, and accurate high-performance liquid chromatography coupled with tandem mass spectrometry (HPLC-MS/MS) method was developed by Design of Experiments (DoE) using a pentafluorophenyl stationary phase. This method allowed the selective quantification of individual 11-keto-boswellic acids and provided evidence for α-KBA in *Boswellia* spp. oleogum resins. The contents of α-KBA as well as further boswellic acids and the composition of essential oils were used to chemotaxonomically classify 41 *Boswellia* oleogum resins from 9 different species. Moreover, α-KBA exhibited cytotoxicity against three treatment-resistant triple-negative breast cancer (TNBC) cell lines in vitro and also induced apoptosis in MDA-MB-231 xenografts in vivo. The respective β-isomer and the acetylated form demonstrate higher cytotoxic efficacies against TNBC cells. This provides further insights into the structure-activity relationship of boswellic acids and could support future developments of potential anti-inflammatory and antitumor drugs.

## 1. Introduction

**Figure 1:**
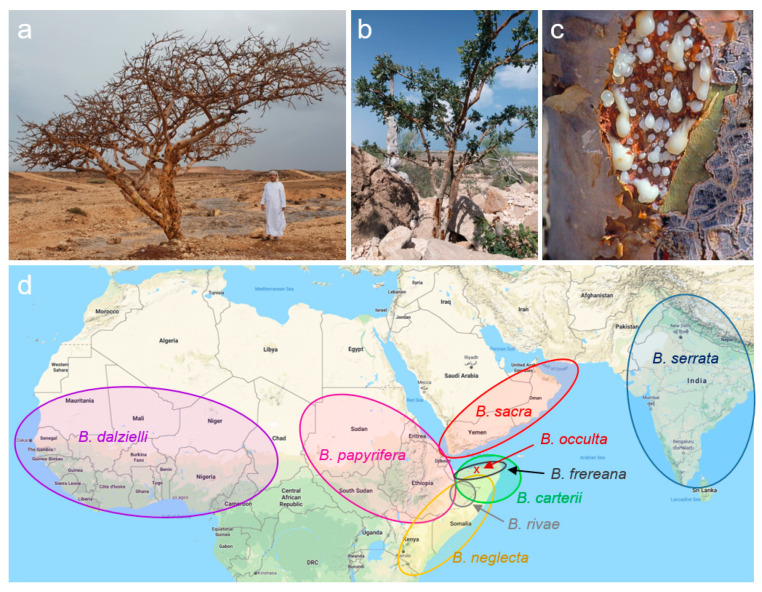
*Boswellia* trees and their areas of distribution. (**a**) *B. sacra* tree growing in the dry regions of Oman. (**b**) Trees of the species *B. frereana* prefer rocky terrains of Somalia. (**c**) Production of frankincense, the oleogum resin of *Boswellia* trees. (**d**) The growth regions of *Boswellia* spp. extend from West Africa to India. A particular biodiversity prevails at the Horn of Africa, in and near Somalia. Pictures reproduced with permission of Georg Huber [13]. Map data ©2020 Google, ORION-ME.

Trees of the genus *Boswellia* Roxb. ex Colebr. (*Burseraceae*) secrete oleogum resins (frankincense), which have been known for centuries for their anti-inflammatory, antibiotic, analgesic, and antitumor activities (Figure 1a–c) [1]. Meanwhile, more than 25 different *Boswellia* species have been described, distributed from India throughout the Arabian Peninsula and the Horn of Africa to West Africa (Figure 1d) [2,3].

Frankincense, the oleogum resin of *Boswellia* spp., is a multicomponent mixture containing 5–15% essential oil, 25–30% ether-insoluble compounds such as polysaccharides, and 55–66% ether-soluble pure resin, containing therapeutically interesting triterpenoids [1,4]. Of particular interest are boswellic acids, which belong to the class of pentacyclic triterpenic acids and are exclusively occurring in the genus *Boswellia* with contents up to 25% [5]. Boswellic acids are also highly abundant in cambium and epidermis, where they are assumed to be synthesized from amyrins [6].

*Boswellia* extracts and boswellic acids have been investigated intensively by modern medicine. Their potential therapeutic efficacy against several chronic inflammatory diseases such as bronchial asthma, rheumatoid arthritis, Crohn’s disease, collagenous colitis, psoriasis, and osteoarthritis was addressed in promising clinical pilot studies [3]. Furthermore, *Boswellia* extracts and boswellic acids induce apoptosis in several cancer cell lines, e.g., from brain cancer, colon cancer, leukemia, and prostate cancer [7]. Here, boswellic acids interact with human topoisomerases (TOP-I/IIα) and the proinflammatory enzyme 5-lipoxygenase (5-LOX), molecular targets for cancer therapy [8,9]. Moreover, boswellic acids inhibit the expression of proinflammatory and prosurvival proteins and tumor-related growth factors by suppressing the activation of the transcription factor NF-κB (nuclear factor kappa-light-chain-enhancer of activated B cells) [10,11,12].

**Figure 2:**
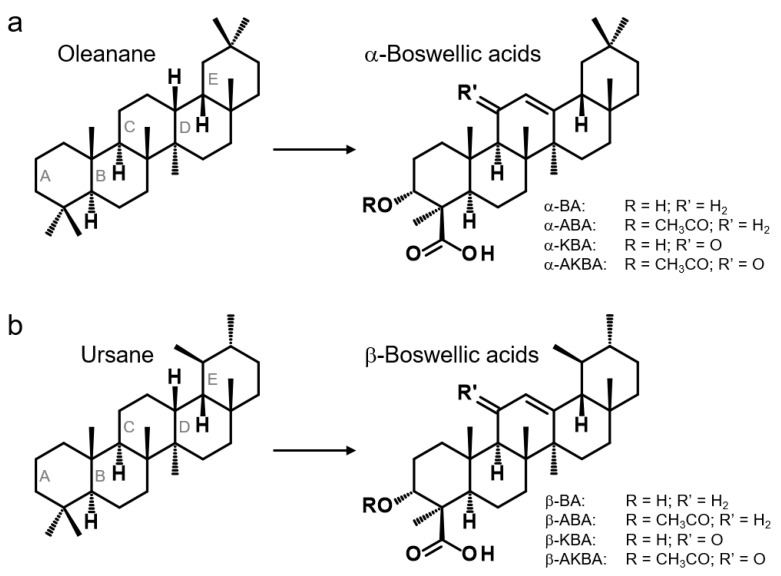
Chemical structures of boswellic acids. (**a**) α-Boswellic acids are based on the oleanane structure: α-boswellic acid (α-BA), acetyl-α-boswellic acid (α-ABA), 11-keto-α-boswellic acid (α-KBA), and acetyl-11-keto-α-boswellic acid (α-AKBA). (**b**) In contrast, β-boswellic acids are derived from the ursane structure: β-boswellic acid (β-BA), acetyl-β-boswellic acid (β-ABA), 11-keto-β-boswellic acid (β-KBA), and acetyl-11-keto-β-boswellic acid (β-AKBA). Corresponding boswellic acids are constitutional isomers, only differing by the position of two methyl groups at ring E. For α-boswellic acids, the methyl groups at ring E are arranged in geminal position, whereas β-boswellic acids have vicinal methyl groups.

Boswellic acids exist in two different structural types: α-boswellic acids, based on the oleanane structure, and β-boswellic acids, derived from the ursane structure (Figure 2) [14]. Hence, the corresponding boswellic acids represent constitutional isomers. It was assumed that the pharmacologically interesting keto-boswellic acids, 11-keto-β-boswellic acid (β-KBA, or commonly abbreviated as KBA) and acetyl-11-keto-β-boswellic acid (β-AKBA, commonly abbreviated as AKBA), only occur as β-isomers [3,7,15,16]. However, in 2005, 70 years after the discovery of the first boswellic acids, we could demonstrate that in addition to β-AKBA, the α-isomer acetyl-11-keto-α-boswellic acid (α-AKBA) naturally occurs in *Boswellia* oleogum resins [17,18]. As a result, it was hypothesized that also the deacetylated form, 11-keto-α-boswellic acid (α-KBA), might exist, because biosynthesis of α-AKBA takes place by acetylation of the precursor α-KBA [6]. In this study, we demonstrate for the first time the existence and the natural occurrence of α-KBA by using a novel, highly selective high-performance liquid chromatography coupled with tandem mass spectrometry HPLC-MS/MS method with a pentafluorophenyl (PFP) stationary phase.

The botanic and taxonomic assignment of a single *Boswellia* specimen is often hampered by overlapping growth regions, but also by geopolitically unstable conditions within the producing countries [1,2,19]. Besides an outstanding genotyping, a chemotaxonomical classification on the basis of secondary metabolites could provide a promising approach to distinguish between the *Boswellia* species. Therefore, the individual contents of α-KBA, β-KBA, α-AKBA, and β-AKBA, as well as the isomer’s ratio, were investigated in 41 oleogum resins of nine different *Boswellia* species. The keto-boswellic acid contents, and additionally the contents of further boswellic and lupeolic acids, and individual essential oil’s compositions, were used to explore the ability for chemotaxonomical classification of different *Boswellia* species.

Furthermore, α-KBA and additional keto-boswellic acids were investigated in terms of cytotoxicity against treatment-resistant triple-negative breast cancer (TNBC) cell lines in vitro and in vivo. With 30%, breast cancers represented the most common cancer type of all new female cancer cases in 2019, causing 15% of all female cancer deaths [20]. Especially, triple-negative breast cancer (TNBC) is a particularly aggressive and highly metastatic subtype, which makes up to 15% of all breast cancer cases and affects mainly younger women [21]. TNBC shows the worst prognosis compared to other breast cancer types, because of the lack of three therapy-relevant receptors, the estrogen receptor (ER), the progesterone receptor (PR), and the human epidermal growth factor receptor 2 (HER2) [22]. The current study aims to provide information about the structure–activity relationship of keto-boswellic acids, assisting future development of potential new anticancer drugs.

## 2. Results and Discussion

### 2.1. Synthesis and Characterization of 11-Keto-α-Boswellic Acid (α-KBA)

To develop an analytical method for selective quantification of individual keto-boswellic acids in *Boswellia* oleogum resins, initially, 11-keto-α-boswellic acid (α-KBA, syn. (3α,4β)-3-hydroxy-11-oxo-olean-12-en-24-oic acid) was synthesized and characterized. Starting from acetyl-α-boswellic acid (α-ABA), firstly, acetyl-11-keto-α-boswellic acid (α-AKBA) was synthesized by radical-type reaction with bromine and characterized as previously described [17]. Afterwards, α-KBA was synthesized by saponification of α-AKBA, with subsequent purification by semi-preparative reversed-phase HPLC.

The compound was obtained as white crystals (m.p. 172–173 °C). Its molecular formula was determined as C_30_H_46_O_4_ by high-resolution electrospray ionization mass spectrometry (HR-ESI-MS) with an exact mass at *m/z* 469.3323 for [M-H]^−^ (calculated for [C_30_H_46_O_4_-H]^−^: 469.3323; error: –0.143 ppm). Investigation of the compound´s isotopic mass pattern corresponded well to the calculated pattern of the formula (see Supplementary Materials Figure S1). Collision-induced fragmentation (CID) of the precursor ion *m/z* 469.3 ([M-H]^−^) by electrospray ionization tandem mass spectrometry (ESI-MS/MS) exhibited characteristic fragments at *m/z* 353.3, 376.3, 391.4, 407.4, and 451.4 (Supplementary Figure S2).

**Figure 3:**
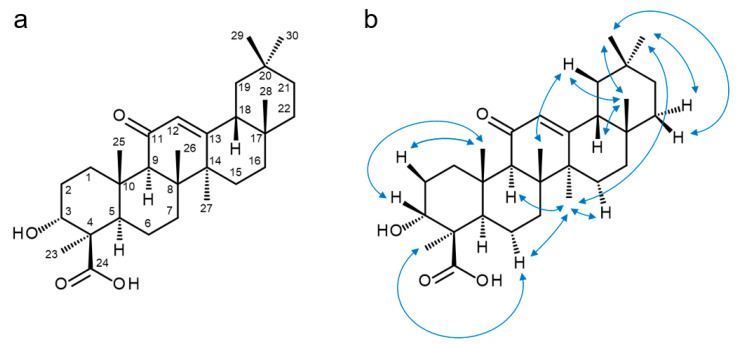
Chemical structure of 11-keto-α-boswellic acid (α-KBA). (**a**) Structure and numbering scheme. (**b**) Illustration of important ROESY (rotating frame Overhauser enhancement spectroscopy) correlations.

Using one- and two-dimensional nuclear magnetic resonance (NMR) spectroscopy, the structure of α-KBA was elucidated (Figure 3a and Supplementary Figures S3–S8). The ^1^H NMR spectrum of α-KBA showed seven singlet signals (CH_3_-23, CH_3_-25, CH_3_-26, CH_3_-27, CH_3_-28 CH_3_-29, and CH_3_-30) accounting for methyl groups attached to quaternary carbons (Table 1). Characteristic for α-boswellic acids, which are derived from oleanane, are two germinal methyl groups at C-20 [14]. In α-KBA, these two geminal methyl groups appeared at δ_H_ 0.88 (CH_3_-29) and δ_H_ 0.89 (CH_3_-30). The presence of a keto function at C-11 (δ_C_ 198.9) was confirmed by long-range heteronuclear correlations with two methine signals, namely the adjacent angular H-9 (δ_H_ 2.35) and the olefinic H-12 (δ_H_ 5.47), which represents the most downfield signal in the ^1^H NMR spectrum. The remaining signals were assigned by homo- and hetero-nuclear correlated NMR spectroscopy, ^1^H, ^1^H COSY (correlated spectroscopy), ^1^H, ^13^C HSQC (heteronuclear single quantum coherence spectroscopy), and ^1^H, ^13^C HMBC (heteronuclear multiple bond correlation). The stereochemistry was elucidated based on correlations in the ^1^H, ^1^H ROESY (rotating frame Overhauser enhancement spectroscopy) spectrum (Figure 3b). Spectral overlaps could be clarified using ^1^H, ^1^H SELTOCSY (selective total correlation spectroscopy), which served as projections for two-dimensional (2D) spectra.

### 2.2. Method Development for Chromatographic Separation of Constitutional Isomers of Keto-Boswellic Acids

For selective and simultaneous quantification of the constitutional isomers of 11-keto-boswellic acids (KBAs), i.e., 11-keto-α-boswellic acid (α-KBA) and 11-keto-β-boswellic acid (β-KBA), as well as acetyl-11-keto-boswellic acids (AKBAs), i.e., acetyl-11-keto-α-boswellic acid (α-AKBA) and acetyl-11-keto-β-boswellic acid (β-AKBA), a novel chromatographic separation method was developed.

**Figure 4:**
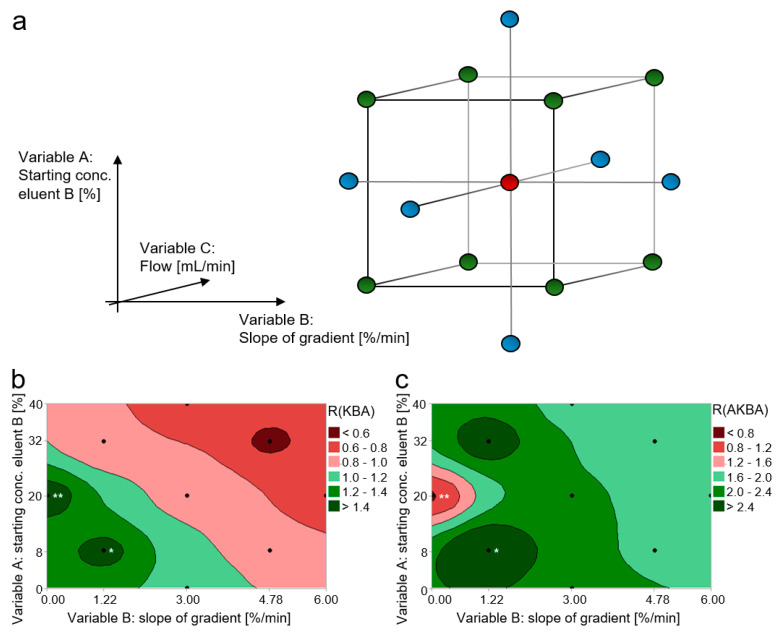
Design of Experiments (DoE) for the development of a chromatographic method to separate the constitutional isomers of keto-boswellic acids (KBAs) and acetyl-keto-boswellic acids (AKBAs). (**a**) Three-factorial central composite design (CCD) with the starting concentration of eluent B as variable A, the slope of the gradient as variable B, and the eluent´s flow as variable C. Whereby, the central composite design is composed of a full two-level factorial (green dots), axial points (blue dots), and a center point (red dot). (**b**) Effect of variables A and B on the chromatographic resolution R(KBA) between 11-keto-α-boswellic acid (α-KBA) and 11-keto-β-boswellic acid (β-KBA). (**c**) Effect of variables A and B on the chromatographic resolution R(AKBA) between acetyl-11-keto-α-boswellic acid (α-AKBA) and acety-11-keto-β-boswellic acid (β-AKBA). Black dots represent the level configurations of the individual experiments. Only the level configurations of experiment 5 (*) enabled a separation of all four isomers. The level configurations of experiment 11 (**) allowed the separation of KBA´s isomers, but the isomers of AKBA could not be eluted under these conditions.

Since the chromatographic separation of the constitutional isomers of KBAs and AKBAs with the help of C18 phases was insufficient [4,5,16,23,24,25,26,27], other stationary phases with varying selectivities were investigated. Here, on the basis of our previous study, a fluorinated stationary phase with pentafluorophenyl (PFP) moieties was used [18]. To achieve sufficient separation efficiency, the chromatographic parameters were optimized by means of Design of Experiments (DoE). As experimental design, a three-factorial central composite design (CCD) was used, with the starting concentration of eluent B as variable A, the slope of the gradient as variable B, and the flow rate as variable C. Hence, the experimental design was composed of a full two-level factorial design (Figure 4a: green dots and Table 2: Experiments 1–8), axial points (Figure 4a: blue dots and Table 2: Experiments 9–14), and a center point (Figure 4a: red dot and Table 2: Experiment 15).

The chromatographic resolution, *R*, between the individual constitutional isomers served as dependent response variables. *R* was calculated with the following formula:

(1) $$R=1.18\times \;\frac{t_{R}\left(\mathrm{Peak}\;2\right)-{\;t}_{R}\left(\mathrm{Peak}\;1\right)}{w_{0.5}\left(\mathrm{Peak}\;2\right)\;+{\;w}_{0.5}\left(\mathrm{Peak}\;1\right)\;}$$

where t_R_ is the respective retention time and w_0,5_ is the respective peak width at half-height. Whereby, two peaks with a resolution *R* ≥ 1.5 were considered as sufficiently separated for individual quantification [28]. To enable a sensitive and accurate detection of the analytes, tandem mass spectrometry in multiple reaction monitoring mode (MRM) was used.

Evaluation of the effects demonstrated that only variables A and B had a significant effect on the chromatographic resolution of α-KBA/β-KBA and α-AKBA/β-AKBA. The experimental design has shown that the level conditions of experiments 5 and 11 enabled a successful chromatographic separation of α-KBA and β-KBA (Figure 4b). However, under the conditions of experiment 11, no sufficient elution of α-AKBA and β-AKBA from the column was possible (Figure 4c).

**Figure 5:**
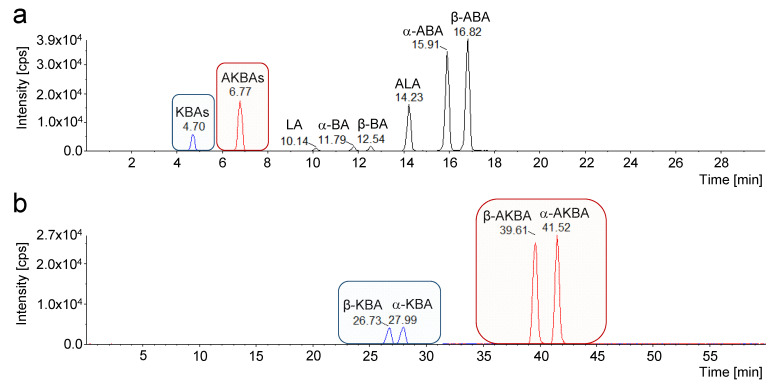
Chromatographic separation of 11-keto-boswellic acids with different stationary phases. (**a**) Multiple reaction monitoring (MRM) chromatogram using a C18 stationary phase. The constitutional isomers lupeolic acid (LA), α-boswellic acid (α-BA), and β-boswellic acid (β-BA), as well as acetyl-lupeolic acid (ALA), acetyl-α-boswellic acid (α-ABA), and acetyl-β-boswellic acid (β-ABA), could be separated sufficiently. However, it is not possible to separate the constitutional isomers of 11-keto-boswellic acid (KBAs) and acetyl-11-keto-bowellic acid (AKBAs) under these conditions [5]. (**b**) MRM chromatogram using a pentafluorophenyl (PFP) stationary phase. The utilization of a PFP stationary phase and optimization of the chromatographic parameters by DoE enabled a successful separation and selective quantification of all KBAs and AKBAs isomers, namely 11-keto-α-boswellic acid (α-KBA), 11-keto-β-boswellic acid (β-KBA), acetyl-11-keto-α-boswellic acid (α-AKBA), and acetyl-11-keto-β-boswellic acid (β-AKBA).

The optimization of the chromatographic parameters by CCD experimental design allowed an investigation of three independent variables (factors) at five levels with only *N* = 2^3^ + 2 x 3 + 1 = 15 experiments, while a full-factorial design with three variables would require *N* = 5^3^ = 125 experiments [29]. Furthermore, the PFP stationary phase provided enhanced dipole–dipole interactions, π–π interactions, charge–transfer interactions, and ion–exchange interactions compared to C18 or C8 phases [30,31]. Hence, the developed novel analytical method, based on experiment 5, enabled a selective quantification of α-KBA, β-KBA, α-AKBA, and β-AKBA for the first time (Figure 5b).

### 2.3. Selective Quantification of 11-Keto-Boswellic Acids (KBAs) and Acetyl-11-Keto-Boswellic Acids (AKBAs) in Oleogum Resins of Boswellia spp.

By means of the novel developed HPLC-MS/MS method, the contents of 11-keto-α-boswellic acid (α-KBA), 11-keto-β-boswellic acid (β-KBA), acetyl-11-keto-α-boswellic acid (α-AKBA), and acetyl-11-keto-β-boswellic acid (β-AKBA) were quantified in 41 oleogum resins of nine different *Boswellia* species. To begin with, the analytical method was validated in terms of linearity, precision, accuracy, recovery, limit of detection (LOD), and limit of quantification (LOQ) (see Appendix Table A1 and Table A2).

Initially, the oleogum resins were extracted exhaustively and analyzed by HPLC-MS/MS with a C18 column, as described previously, to obtain total contents of 11-keto-boswellic acids (KBAs) and acetyl-11-keto-boswellic acids (AKBAs), respectively [5]. The analysis with C18 stationary phases enables chromatographic separation of several boswellic acids, including respective constitutional isomers, but so far, without sufficient separation of keto-boswellic acid constitutional isomers (Figure 5a) [4,5,16,23,24,25,26,27]. However, by using a pentafluorophenyl (PFP) stationary phase and the optimized chromatographic parameters, selective analysis of the constitutional isomers of KBAs and AKBAs, i.e., α-KBA, β-KBA, α-AKBA, and β-AKBA, was achieved (Figure 5b). Thus, it could be demonstrated for the first time, that besides the well-known β-KBA, the hitherto unknown α-isomer, α-KBA, naturally occurs in *Boswellia* spp.

The analyses of 41 *Boswellia* samples showed that with 1.1–7.8%, α-KBA makes only a small proportion of the total KBAs in oleogum resins of *B. sacra*, *B. dalzielli*, *B. papyrifera*, *B. serrata*, or *B. occulta* (Table 3). Similarly, α-AKBA makes only 0.5–6.4% of the total AKBAs in those species. In contrast, in oleogum resins of *B. carterii* and *B. neglecta*, the proportion of α-KBA was much higher, reaching 25.5% and 32.9%, respectively. Likewise, the proportions of α-AKBA were increased with maximum amounts of 14.8% in *B. carterii* and 18.4% in *B. neglecta*. Remarkably, the oleogum resin of *B. rivae* was the only sample that exhibited a higher proportion of α-KBA (57.7%) than β-KBA. In oleogum resins of the species *B. frereana*, no KBAs and AKBAs could be detected. This corresponds to previous studies, which showed the lack of boswellic acids in this species [5,24]. Likewise, in one sample of *B. neglecta* from Kenya, the contents of KBAs were below the LOQ, too.

In general, the absolute contents of α-KBA in frankincense was rather low, with a maximum concentration of 1.0 µg per mg oleogum resin. Though, the total content of KBAs of up to 22.3 µg/mg represents an unneglectable part of the oleogum resins. Similarly, the maximum content of α-AKBA was with 2.8 µg/mg rather low, whereas the total amount of AKBAs can reach up to 72.2 µg/mg [5].

### 2.4. Investigation of Essentials Oils from the Oleogum Resins of Boswellia spp. 

To investigate the contents of KBAs and AKBAs as well as the compositions of mono- and di-terpenes in essential oils from *Boswellia* spp., essential oils were prepared by hydrodistillation of *Boswellia* oleogum resins. Hydrodistillation yielded 9.7% essential oil in *B. sacra* (*w/w*), 1.5% essential oil in *B. serrata* (*w/w*), 5.1% essential oil in *B. carterii* (*w/w*), 5.9% essential oil in *B. frereana* (*w/w*), 9.0% essential oil in *B. dalzielli* (*w/w*), 1.2% essential oil in *B. papyrifera* (*w/w*), 3.3% essential oil in *B. neglecta* (*w/w*), and 3.3% essential oil in *B. rivae* (*w/w*).

HPLC-MS/MS analysis exhibited contents of KBAs below LOQ in all essential oil samples. Likewise, the contents of AKBAs in essential oils of *B. sacra*, *B. serrata*, *B. carterii*, *B. frereana*, and *B. dalzielli* were below LOQ. Only in essential oils of *B. papyrifera*, *B. neglecta*, and *B. rivae* could very low concentrations of 2–3 ng/mg AKBAs be found. Given the high molecular weight of boswellic acids, these results are to be expected. Hence, triterpenes are not recognized to be usual constituents of distilled essential oils.

Gas chromatographic (GC) analysis showed that essential oils consist mainly of the monoterpenoids α-thujene, α-pinene, β-pinene, sabinene, *para*-cymene, and limonene (Table 4). This generally complies with previously published data, where different *Boswellia* essential oils were investigated [32,33]. Thus, our data illustrate that essential oils from *B. sacra* and *B. carterii* are chemically distinct, with α-pinene being more dominant in the former species [33], despite the fact that *B. carterii* is often considered a synonym of *B. sacra* (see Section 2.5.5). In contrast to all other samples, the main component of *B. serrata* essential oil was myrcene, with 41.4%. Such a high content of myrcene in *B. serrata* is quite unusual, since the main monoterpene of *B. serrata* essential oil is frequently α-thujene [34], but high myrcene contents have been already observed in other studies [32,35]. Notably, high amounts of myrcene might not be harmless because the International Agency for Research on Cancer (IARC) classified it in 2017 as a potential carcinogen [36,37]. However, this classification is currently under discussion [38]. Furthermore, essential oil from *B. serrata* contained 6.0% methylchavicol (estragole), which is under suspicion as a genotoxic compound [39,40].

Interestingly, all essential oil samples contained the monoterpene 5,5-dimethyl-1-vinylbicyclo[2.1.1]hexane (hashishene). Hashishene is formed by photo-oxidation of myrcene and was given its common name from hashish, resins of *Cannabis sativa* [41]. As in hashish, a similar formation of hashishene by solar radiation is conceivable during the harvest of *Boswellia* resins. Particularly, the essential oil from *B. serrata* investigated in this study exhibits the highest amount of myrcene and as a result, the highest amount of hashishene.

### 2.5. Chemotaxomic Classification of Boswellia spp. on the Basis of Boswellic Acid and Essential Oil Compositions

As the distribution areas of the more than 25 known *Boswellia* species overlap and taxonomic specifications are rarely clearly defined, the botanic assignment of individual specimen has always been hampered [1]. This is particularly true for the biodiversity hotspot located at the Horn of Africa, where most *Boswellia* species flourish [2,42]. Furthermore, an on-site classification is often difficult, because the habitat regions are mostly located in less developed and geopolitically unstable countries, e.g., Somalia or Yemen. Hence, a chemotaxonomic classification on the basis of phytochemicals such as boswellic acids could represent an interesting alternative. Hereinafter, the contents of KBAs and AKBAs, the essential oil´s compositions, as well as further data will be discussed as typical for individual *Boswellia* species.

#### 2.5.1. *Boswellia sacra* Flueck.

The species *B. sacra* is mostly distributed at the Arabian Peninsula (Oman and Yemen), but occasionally, there have also been specimens described in Somalia (Figure 1a) [1]. Frankincense from *B. sacra* is considered as the most valuable and, as a result, the most expensive one [19].

**Figure 6:**
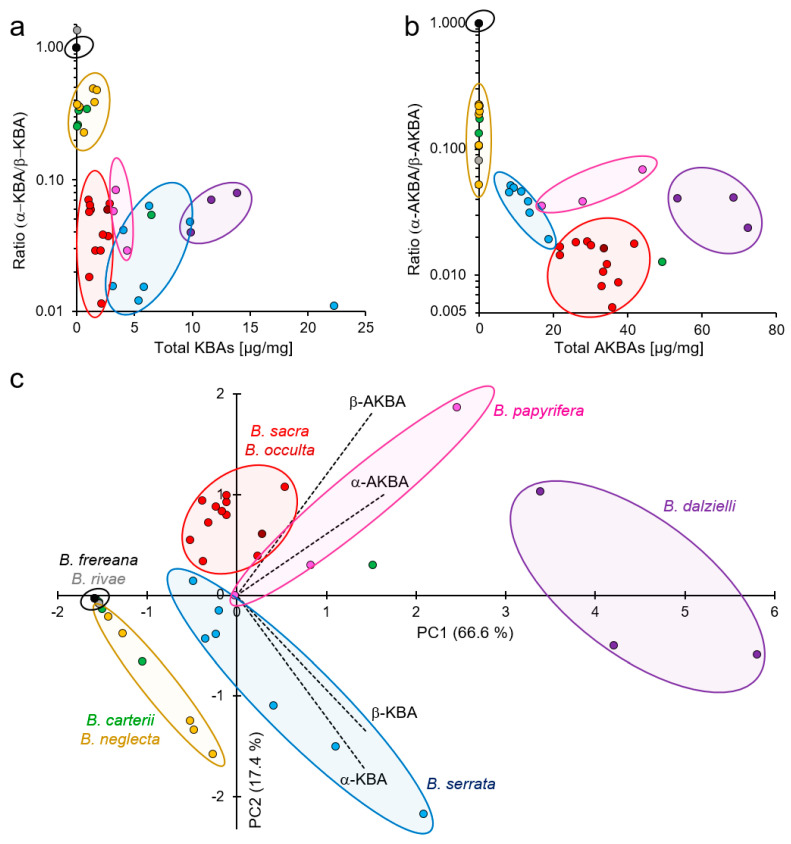
Visualization of variations in composition of 11-keto-boswellic acids (KBAs) and acetyl-11-keto-boswellic acids (AKBAs) in oleogum resins of different *Boswellia* species. *B. sacra* (red), *B. serrata* (blue), *B. dalzielli* (violet), *B. papyrifera* (pink), *B. carterii* (green), *B. neglecta* (yellow), *B. frereana* (black), *B. rivae* (grey), and *B. occulta* (dark red). (**a**) Semi-logarithmic scatter plot of total KBA contents and ratio between 11-keto-α-boswellic acid (α-KBA) and 11-keto-β-boswellic acid (β-KBA). (**b**) Semi-logarithmic scatter plot of total AKBA contents and ratio between acetyl-11-keto-α-boswellic acid (α-AKBA) and acetyl-11-keto-β-boswellic acid (β-AKBA). Contents < LOQ were considered as 0.000 µg/mg and as constitutional isomers ratio of 50/50. (**c**) Biplot of principal component analysis (PCA) with loadings of α-KBA, β-KBA, α-AKBA, and β-AKBA (dashed lines), and individual scores of samples (dots).

Assessing the KBAs composition, *B. sacra* exhibited a unique combination of low total content KBAs (1.0–2.8 µg/mg) and coincidently, a low proportion of α-KBA (1.1–6.6%) (Figure 6a). Furthermore, *B. sacra* also showed a low proportion of α-AKBA (0.5–1.8%), but relatively high total contents of AKBAs with 21.7–41.8 µg/mg (Figure 6b). This complies with previous studies that observed a high percentage of acetylated boswellic acids in *B. sacra* oleogum resins [5,16].

#### 2.5.2. *Boswellia serrata* Roxb. ex. Colebr.

Trees of *B. serrata* (syn. *B. thurifera* Colebr. and *B. glabra* Roxb.) grow in the central and northern parts of India and in Pakistan [19]. The geographical delineation by the Arabian Sea isolates *B. serrata* from other *Boswellia* species [1]. Frankincense from *B. serrata* represents probably the best investigated *Boswellia* oleogum resin and it has been used in Ayurvedic medicine for centuries [3,7,15,43].

The total KBA contents of *B. serrata* differed from those of *B. sacra* with higher values of 3.0–22.9 µg/mg. The proportion of α-KBA was low. In contrast to *B. sacra*, *B. serrata* contained lower total AKBA contents (8.0–18.7 µg/mg). Thus, this confirms previous results showing that the composition of oleogum resins from *B. serrata* differ from that of *B. sacra* due to a high percentage of deacetylated boswellic acids [5,16]. This dissimilarity becomes especially apparent by the different score positions within the biplot of the principal component analysis (Figure 6c). The unique boswellic acid composition in combination with the occurrence of methylchavicol is characteristic for *B. serrata* oleogum resins.

#### 2.5.3. *Boswellia papyrifera* Hochst.

The growth region of *B. papyrifera* is Ethiopia, Eritrea, and Sudan [19]. Frankincense from *B. papyrifera* is mostly used as incense in churches and it is potentially endangered by over-harvesting [2].

Although *B. papyrifera* exhibits a similar boswellic acid composition compared to *B. sacra* [5], the KBAs and AKBAs analysis revealed differences. Oleogum resins of *B. papyrifera* contained higher total KBAs contents (3.1–4.8 µg/mg) and a higher proportion of α-AKBA (3.4–6.4%) than *B. sacra*.

Furthermore, GC analysis of the essential oil revealed exceptionally high amounts of octanol (7.0%), octanol acetate (77.7%), and the potential psychoactive [44,45] diterpenoid incensole (0.05%). This unique feature has been confirmed by several studies and makes *B. papyrifera* clearly distinguishable from other *Boswellia* species [32,46].

#### 2.5.4. *Boswellia dalzielli* Hutch.

Unlike other *Boswellia* species, *B. dalzielli* (syn. *B. dalzielii*) is distributed in West and Central Africa, especially in Nigeria, Senegal, and Burkina Faso [1,19]. In traditional African medicine, it is primarily the bark of the up to 13 m high *B. dalzielli* trees that is used [47].

At first glance, oleogum resins of *B. papyrifera* resemble those of *B. sacra* with regard to the contents of acetylated boswellic acids [5] and of α-pinene (Table 4). However, a closer look at the contents of KBAs and especially AKBAs reveals that *B. dalzielli* is unique (Figure 6c). The total AKBAs content (53.4–72.2 µg/mg) is significantly higher compared to all other species (*p* < 0.01, one-way analysis of variance (ANOVA) post hoc Fisher´s least significant difference (LSD), whereby the proportion of α-AKBA is higher too compared to *B. sacra*. Furthermore, the total KBAs content (9.8–13.8 µg/mg) is higher than in most other *Boswellia* species.

#### 2.5.5. *Boswellia carterii* Birdw.

*B. carterii* (syn. *B. carteri* or *B. bhaw-dajiana* Birdw.) thrives in North Somalia, especially in the regions of Bari, Puntland, and Somaliland [1,19]. Its oleogum resin represents the region´s key exports, its incense serves the indigenous population as a natural insecticide [19]. *B. carterii* is a prime example for the difficulty of taxonomic and botanic classification of *Boswellia* species. After the discovery of *B. sacra* in 1867 by Friedrich August Flückiger, George Birdwood redefined the species as *B. carterii* in 1870 [1,48]. In 1969, Frank Nigel Hepper differentiated this species into Arabian *B. sacra* Flueck. and African *B. carterii* Birdw. [49]. Until now, botanists and other scientists are still discussing whether *B. sacra* and *B. carterii* are the same or two different species [16,24,26,33,42,50].

Analyses of KBAs contents revealed that four of five *B. carterii* samples contained a very low total KBA content (0.08–0.87 µg/mg) with a relatively high proportion of α-KBA (20.3–25.5%) (Figure 6a). Likewise, the total AKBA content of these samples was very low (0.001–0.1 µg/mg), whereas the α-AKBA proportion (9.7–14.8%) was enhanced (Figure 6b). However, one *B. carterii* sample exhibited higher total KBAs and AKBAs contents with a lower proportion of the α-isomers but was still distinguishable from *B. sacra* samples (Figure 6c). Moreover, GC analysis of the essential oil of *B. carterii* revealed a lower content of α-pinene, but higher contents of inter alia limonene, α-thujene, *para*-cymene, and α-phellandrene compared to *B. sacra* (Table 4). A study by Wooley et al., comparing essential oils from *B. carterii* and *B. sacra*, yielded the same results and revealed additionally major differences in enantiomeric ration of α-pinene [33]. Furthermore, a comparative analysis of the boswellic and lupeolic acid compositions showed clear differences, too, between *B. carterii* and *B. sacra* samples [5]. In conclusion, the analyzed *B. carterii* and *B. sacra* samples were clearly distinguishable, suggesting that they are two different species. Genotyping could provide further evidence.

#### 2.5.6. *Boswellia frereana* Birdw.

The species *B. frereana* is located in Northern Somalia and in contrast to all other *Boswellia* species, its trees prefer rocky terrain (Figure 1b) [1]. Frankincense obtained from *B. frereana* is golden yellow and of an extraordinary tender consistence and is frequently chewed by the indigenous population [19].

*B. frereana* is the only *Boswellia* species that contains no boswellic or lupeolic acids [5,24]. Accordingly, the contents of KBAs and AKBAs were below the LOQ and LOD in this study, too. This fact makes *B. frereana* unique and clearly distinguishable from all other *Boswellia* species. Interestingly, *B. frereana* contains high amounts of lupeol and epi-lupeol [24], which are precursors for boswellic acid biosynthesis [6]. Hence, a possible explanation for the lack of boswellic acids might be a missing gene of the oxidoreductase necessary for formation of the carboxyl functional group.

#### 2.5.7. *Boswellia occulta* Thulin, DeCarlo & S. P. Johnson

In the biodiversity hotspot of Somalia, *B. occulta*, a novel *Boswellia* species, was recently discovered. Trees of this species are only distributed in a small area in northwestern Somalia and show high similarity to *B. sacra* [51].

Likewise, total contents of KBAs and AKBAs as well as the individual isomer proportion in *B. occulta* were indistinguishable from those of *B. sacra* (Figure 6a, b, c). Moreover, the contents of further boswellic and lupeolic acids in oleogum resins of *B. occulta* and *B. sacra* are very similar [5]. However, analysis of the essential oils of *B. occulta* revealed an outstandingly high amount of methyl esters, especially 1-methoxydecane, which was identified so far as a major constituent only for this species [52,53].

#### 2.5.8. *Boswellia neglecta* S. Moore and Boswellia rivae Engl.

*B. neglecta* from Kenya produces very dark, hard-brittle frankincense called *Dakkara*, while *B. neclecta* from Somalia yields yellow golden to dark grey frankincense called *Muqlo*, *Gunra*, and *Mirafur* [19,54]. The species *B. rivae* is distributed in the politically unstable region Ogaden. The frankincense of *B. rivae* has a dark grey or brown appearance and hard-brittle consistency [19].

*B. neglecta* olegum resins from Somalia were very similar to *B. carterii* with low total contents of KBAs (0.2–1.7 µg/mg) and AKBAs (0.03–0.14 µg/mg) and high proportions of the respective α-isomers, α-KBA (18.5–32.9%) and α-AKBA (15.9–18.4%). Considering the similar contents of further boswellic and lupeolic acids, the differentiation between *B. neglecta* and *B. carterii* species is hampered. Only the high contents of acetyl-lupeolic acid, acetyl-α-boswellic acid, and acetyl-β-boswellic acid in some *B. carterii* gum resins might serve as a reference point for differentiation [5].

Differently, oleogum resins of *B. neglecta* from Kenya as well as *B. rivae* are characterized by very low total contents of KBAs (<LOQ 0.06 µg/mg) and AKBAs (0.002–0.055 µg/mg). Remarkably, *B. rivae* is the only sample that exhibited a higher proportion of the α-isomer α-KBA with 57.7%, which makes it unique. Further investigations of *B. neglecta* (from Kenya) and *B. rivae* revealed very low contents of all boswellic and lupeolic acids [5]. It has been reported that at harvesting of these species, the oleogum resins can be mixed with olegum resins from genus *Commiphora*, such as the rather unknown species *Commiphora confusa* [19,54]. Analysis of *Commiphora* specific compounds, e.g., *E*- and *Z*-guggulsterone, could provide further information on that.

### 2.6. Cytotoxic Efficacy of 11-Keto-α-Boswellic Acid (α-KBA) against Triple-Negative Human Breast Cancer Cells In Vitro and In Vivo

**Figure 7:**
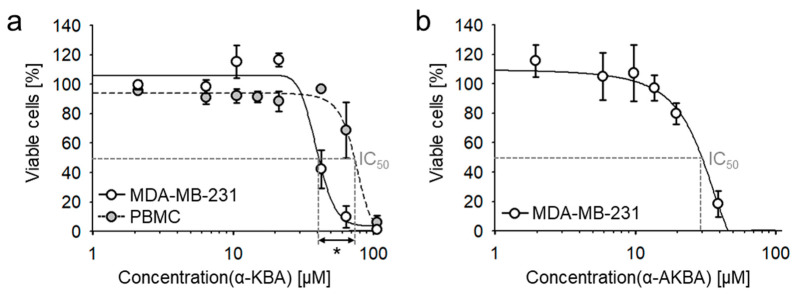
Cytotoxicity of 11-keto-α-boswellic acid (α-KBA) and acetyl-11-keto-α-boswellic acid (α-AKBA) against the human triple-negative breast cancer cell line MDA-MB-231 in vitro. (**a**) Comparison of the cytotoxic efficacies of α-KBA against MDA-MB-231 cells and human peripheral blood mononuclear cells (PBMC). Cancer cells were more sensitive to α-KBA than non-cancerogenic PBMC. (**b**) The acetylated form, α-AKBA, exhibits higher cytotoxicity compared to α-KBA. XTT (2,3-bis-(2-methoxy-4-nitro-5-sulfophenyl)-2H-tetrazolium-5-carboxanilide salt) assay, 72 h incubation, *n* = 3 (all biological replicates were performed in triplicates). Student´s *t*-test, * *p* < 0.05.

Investigation of cytotoxic efficacy revealed that α-KBA concentration-dependently inhibited the viability of the highly metastatic, treatment-resistant, triple-negative human breast cancer (TNBC) cell line MDA-MB-231 in vitro with a half maximal inhibitory concentration (IC_50_) of 42.0 ± 4.6 µM (Figure 7a). We have previously shown that boswellic acids target transcriptional factor NF-κB in immune cells [10,12]. Hence, the effect of α-KBA on the viability of TBNC cells was compared to that of peripheral blood mononuclear cells (PBMC). Interestingly, cancer cells were statistically significantly (Student´s *t*-test, *p* = 0.031) more sensitive to α-KBA compared to human PBMC, the non-cancerogenic control group (IC_50_ = 75.2 ± 9.0 µM). However, comparing the cytotoxicity of KBA α- and β-isomers, β-KBA is more effective against MDA-MB-231 cells (Table 5). Comparing again the deacetylated and acetylated forms of α-KBA, acetylated α-AKBA exhibited higher cytotoxicity (Figure 7b). In fact, the acetylated β-isomer, β-AKBA, was the most effective one, indicating that β-configuration and acetylation increase the cytotoxic efficacy against TNBC cells. Comparing cytotoxicity against MDA-MB-231 and calculated ALogP values showed increasing efficacy with increasing lipophilicity. This phenomenon can be explained by cellular permeability, which is increased with increased lipophilicity [55].

Further, the cytotoxic efficacy of α-KBA was investigated on two additional TNBC cell lines, CAL-51 and CAL-148. Likewise, α-KBA exhibited considerable cytotoxicity against CAL-51 cells (IC_50_ = 34.6 ± 1.9 µM) and CAL-148 cells (IC_50_ = 36.1 ± 1.9 µM), indicating cytotoxic efficacy against different treatment-resistant TNBC cells.

**Figure 8:**
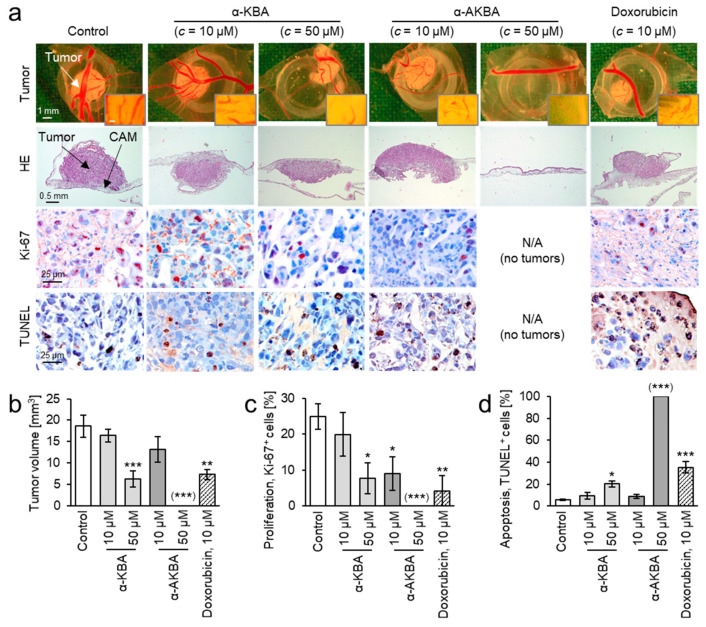
11-keto-α-boswellic acid (α-KBA) and acetyl-11-keto-α-boswellic acid (α-AKBA) inhibit proliferation and tumor growth and induce apoptosis in breast cancer xenografts in vivo. MDA-MB-231 cells were grafted onto the chorioallantoic membrane (CAM) of fertilized chick eggs and treated for 3 consecutive days with either α-KBA (10 and 50 µM), α-AKBA (10 and 50 µM), doxorubicin (10 µM), or DMSO (0.5%) as vehicle control. After treatment with 50 µM α-AKBA, no tumors could be observed. Hence, results for α-AKBA (50 µM) are considered as 0% proliferating cells or 100% apoptotic cells and delineated in brackets. (**a**) 1st row: tumor photographs immediately after extraction (original magnification 50×). Inserts: peritumoral blood vessels (bar: 100 µm). 2nd row: hematoxylin and eosin staining. 3rd row: staining for proliferation marker Ki-67 (red-brown nuclear stain, original magnification 200x). 4th row: TUNEL (terminal deoxynucleotidyl transferase dUTP nick end labeling) staining for apoptosis (brown, original magnification 200x). Representative pictures are shown. (**b**) α-KBA and α-AKBA concentration-dependently inhibit the tumor growth and (**c**) the cancer cell proliferation. (**d**) α-KBA and α-AKBA induce apoptosis in cancer xenografts. All data are mean ± standard error of the mean (SEM), *n* = 4–6. Comparison with control by one-way ANOVA and post hoc by Dunnett´s test with * *p* < 0.05, ** *p* < 0.01, and *** *p* < 0.001.

The antitumor activity of α-KBA was further verified in vivo, by means of MDA-MB-231 breast cancer xenografts grown on the chorioallantoic membrane (CAM) of fertilized chick eggs. Moreover, the influence of acetylation on cytotoxicity in vivo was analyzed. After treatment for 3 consecutive days with the individual compounds at different doses, the tumor volumes were measured and specimens were analyzed immunohistochemically regarding proliferation and apoptosis by Ki-67 antigen staining and the terminal deoxynucleotidyl transferase dUTP nick end labeling (TUNEL) method, respectively (Figure 8a).

Treatment of breast cancer xenografts with 50 µM α-KBA significantly reduced the tumor volume and cancer cell proliferation, and induced apoptosis (Figure 8b–d). However, treatment with 10 µM α-KBA showed no statistically significant effects on breast cancer xenografts. In contrast to α-KBA, α-AKBA inhibited the proliferation of breast cancer cells already at 10 µM treatment. Whereas, the treatment with 50 µM α-AKBA resulted in total absence of tumors. Thus, the in vivo results confirmed that acetylated keto-boswellic acids are more potent against TBNC cells than their deacetylated forms. This corresponds with previously published data showing a high correlation between contents of acetylated boswellic acids in *Boswellia* extracts and their cytotoxicity against MDA-MB-231 cells [5,27]. Moreover, it was demonstrated that acetylated boswellic acids inhibit the activity of human topoisomerases by direct interaction, similar to anticancer drugs such as camptothecin and etoposide [9]. In addition, acetylated boswellic acids suppress the expression of antiapoptotic proteins by inhibiting the activity of the transcriptional factor NF-κB [10,11,12]. Notably, no death or obvious malformation of chick embryos were observed, which points to low systemic toxicity of α-KBA and α-AKBA.

As a positive control for in vitro and in vivo experiments, the non-halogenated anthracycline doxorubicin was analyzed. Doxorubicin is used as a chemotherapeutic agent for the treatment of breast cancer patients. Doxorubicin exhibited higher toxicity against MDA-MB-231 cells in vitro (IC_50_ = 0.71 ± 0.05 µM) [27] and antiproliferative and apoptosis-inducing activity in vivo (Figure 8) than the boswellic acids analyzed in the present study. However, doxorubicin induces severe adverse effects, like cardio- and nephrotoxicity [56,57], whereas, for *Boswellia* extracts and boswellic acids, no adverse effects were observed in mouse studies [11,12,58] and only mild adverse effects such as heartburn or nausea have been reported in clinical studies [3].

The European pharmacopoeia (Ph. Eur.) recommends the characterization of extracts from *B. serrata* by their β-KBA and β-AKBA contents [59]. However, the recommended HPLC method using a C18 column is unable to distinguish the corresponding α- and β-isomers. Furthermore, compared to other boswellic acids, KBAs and particularly AKBAs contents correlated only weakly with cytokine release inhibition and cytotoxicity against MDA-MB-231 cells [27]. Considering that acetyl-β-boswellic acid (β-ABA) exhibited the highest correlation with cytokine inhibition, the highest cytotoxicity against cancer cells, besides being clearly quantifiable [4,5,23,24,26,27], β-ABA should be considered for pharmaceutic standardization of *Boswellia* oleogum preparations.

## 3. Materials and Methods

### 3.1. Material and Samples

All solvents and chemicals were of analytical reagent grade. The solvents used for the extraction, sample preparation, and HPLC-MS/MS analysis were MeOH, acetic acid (both HiPerSolv Chromanorm, VWR chemicals, Fontenay-sous-Bois, France), and ultrapure water (reverse-osmosis type water (pureAqua, Schnaitsee, Germany)) coupled to a Milli-Q station (Millipore, Eschborn, Germany). The compounds 11-keto-β-boswellic acid (β-KBA) and acetyl-11-keto-β-boswellic acid (β-AKBA) were purchased from Extrasynthese (Genay Cedex, France). Acetyl-11-keto-α-boswellic acid (α-AKBA) was synthesized and characterized as previously described [17].

*Boswellia* oleogum resins were purchased from commercial sources or were obtained from cooperation partners. All samples were thoroughly examined and numbered as previously detailed [5]. Samples # 1–8, 12–15, 17–21, and 25–40 were purchased from Georg Huber (Jeomra, Seeheim, Germany). Samples # 22–24 were from Alfred Galke (Alfred Galke GmbH, Bad Grund, Germany). Samples # 9–11 were provided by Prof. Dr. L.J. Rashan (Medical Plant Division, Dhofar University, Salalah, Oman), sample # 16 by Stephan Pohl (Staufen, Germany), and sample # 41 by Prof. Dr. M. Thulin (Evolutionary Biology Centre, Department of Organismal Biology, Uppsala University, Sweden). Voucher specimens of all *Boswellia* samples are deposited at the Herbarium of the Botanical Garden of Ulm University, Institute of Systemic Botany and Ecology, Germany (voucher: ULM-24224). For more precise sample information, please see the previous article and corresponding Supplementary Materials [5].

### 3.2. Synthesis of α-KBA

α-KBA was synthesized by saponification of α-AKBA. For this purpose, 6.99 mg (13.6 µmol) of α-AKBA was dissolved in 420 µL of 10% KOH in MeOH (*m/v*). For base hydrolysis, the solution was heated to 75 °C for 25 min with continuous stirring. The reaction was stopped by adding 420 µL concentrated acetic acid yielding a pH value of 4.5. The reaction product was precipitated by adding 1000 µL warm water and subsequently cooling down for 30 min at 4 °C. Afterwards, the product was washed three times with 1000 µL cold water and dried by lyophilization yielding 5.73 mg raw product.

The raw product was purified by semi-preparative HPLC using a LC-9A HPLC pump (Shimadzu, Kyoto, Japan), an IWN CH100 column oven (Junedis, Gröbenzell, Germany), an UVD 340 U photodiode array detector (Dionex, Idstein, Germany), and a fraction collector (Gilson, Limburg-Offheim, Germany). Instruments were controlled and data processed by Chromeleon software version 6.6 (Dionex, Sunnyvale, CA, USA). For purification, a semi-preparative column (Phenomenex, Synergi Hydro-RP, 4 μm, 80 Å, 250 × 10 mm) with a precolumn (Phenomenex, SecurityGuard, C18, 4 × 3 mm) was used. The raw product was dissolved in 1000 µL DMSO and filtered through a 0.45 µm regenerated cellulose filter. The flow rate was 5000 µL/min and the injection volume was 250 µL. The mobile phase consisted of eluent A, MeOH/water (60/40, *v/v*), and eluent B, MeOH, both acidified with 0.2% acetic acid. Initial conditions were 30% eluent A and 70% eluent B followed by a linear gradient to 90% eluent B over 15 min, then 90% eluent B until 25 min. Thereafter, followed a linear gradient to initial conditions until 25.5 min and re-equilibration continued until 30 min. In order to stabilize the chromatographic system, the column was kept at a temperature of 28 °C.

The collected fractions were concentrated with a nitrogen stream first, and then dried by lyophilization, yielding 3.94 mg (8.37 µmol) α-KBA. This corresponds to a total yield of 61.8% (*n/n*). Purity control by high-performance liquid chromatography with photodiode array detection (HPLC-DAD) at 210, 254, and 280 nm exhibited a purity > 99%.

### 3.3. Charactization and Structure Elucidation of α-KBA

A high-resolution mass spectrum of α-KBA was recorded with a 12 Tesla solariX FT-ICR-MS (Fourier transform ion cyclotron resonance mass spectrometer; Bruker Daltonics, Bremen, Germany) equipped with an APOLLO II electrospray source (Bruker Daltonics) and operated in the negative ionization mode. A diluted sample (*β* = 10 µg/mL in MeOH/water (7/3, *v/v*)) was infused into the MS with a constant flow rate of 120 µL/h. Spectra were acquired with a time-domain of 4 megawords and 30 scans were accumulated within an *m/z* range from 147 to 750. Calibration of the MS and ion source settings were the same as recently described [60]. The MS method achieved highest resolving power of 393,000 at *m/z* 469.332267 and superb mass accuracy in the ppb range.

All NMR (nuclear magnetic resonance) spectra of α-KBA were recorded on a 500 MHz Bruker Avance III HD spectrometer equipped with a CryoPlatform and a 5 mm TCI CryoProbe (Bruker Biospin, Karlsruhe, Germany). Spectrometer control and data processing were accomplished with Bruker TopSpin Ver. 3.6.1. The sample was measured in DMSO-*d_6_* and data were referenced to the remaining solvent signals at δ_H_ 2.50 and δ_C_ 39.51, respectively.

### 3.4. Quantification of 11-Keto-Boswellic Acids and Acetyl-11-Keto-Boswellic Acids by HPLC-MS/MS

All HPLC-MS/MS experiments were carried out on an Agilent 1260 Infinity HPLC system (Agilent, Santa Clara, CA, USA) coupled with an AB API 2000 triple quadrupole mass spectrometer (Applied Biosystem, Foster City, CA, USA) using an electrospray ionization ion source (ESI). Devices were controlled and data were processed by means of Analyst 1.6.1 software (AB Sciex, Framingham, MA, USA).

The quantification of total contents of KBAs and AKBAs was executed by a HPLC-MS/MS method developed, validated, and implemented as previously published [5,27]. To quantify the individual constitutional isomers of KBAs and AKBAs, a novel method was developed by means of Design of Experiments (DoE) with central composite design (CCD). Here, a pentafluorophenyl column (Dr. Maisch, Fluorsil 100 PFP, 3 µm, 100 × 3 mm; Dr. Maisch GmbH, Ammerbruch, Germany) with a C18 precolumn (Dr. Maisch ReproSil Universal RP, 5 µm, 10 × 4 mm) were used. To ensure an orthogonal experimental design, the distances, α, between the center point and the axial points of the star design were calculated with following formula:

(2) α2= 12(N×NC − NC)

where *N* is the total number of experiments and *N_C_* is the number of experiments of the cube, i.e., the full two-level factorial design [29]. The experiments were carried out in a randomized order to avoid falsification of the model by bias. The evaluation of the effects was done with the help of normal probability plots.

The mobile phase consisted of eluent A, MeOH/water (60/40, *v/v*), and eluent B, MeOH, both acidified with 0.2% acetic acid. The injection volume was 20 µL and the column was kept at a stable temperature of 28 °C. On the basis of the results obtained by the DoE, the method herein was developed: Initial conditions were 92% eluent A and 8% eluent B, followed by a linear gradient to 62% eluent B over 44.5 min, and subsequent linear gradient to 95% eluent B until 45.0 min. Thereafter, 95% eluent B until 54.5 min, followed by linear gradient to initial condition until 55.0 min, and re-equilibration until 60.0 min. Here, the flow rate of the mobile phase was 690 µL/min.

The MS/MS analysis was performed in the negative ionization mode with multiple reaction monitoring (MRM) detection. The atmospheric pressure electrospray ion source was heated to 400 °C and the ion spray voltage was set to −4250 V. Collision energy was optimized to −40 V and the dwell time to 250 ms. The precursor ion at *m/z* 469.4 and the product ion with the highest intensity at *m/z* 391.4 were selected for α-KBA and β-KBA. Similarly, the precursor ion at *m/z* 511.4 and the product ion *m/z* 59.0 were used for α-AKBA and β-AKBA.

For sample preparation, the samples were freshly dissolved in MeOH (*β* = 1 mg/mL or *β* = 10 mg/mL). To determine the individual contents, the total content was divided with regard to the ratio of the areas under curves (AUC) of the individual compound’s peak within the MRM chromatograms. To ensure a proportional ratio, the linearity was investigated for each individual compound at 10 concentration levels. Thus, this procedure ensured a linear proportion (*p* < 0.001) between concentration and AUC in a range of 1–1000 ng/mL for α-KBA, β-KBA, α-AKBA, and β-AKBA. All samples were diluted according to this linear range and analyzed in duplicates to ensure an accurate quantification.

### 3.5. Preparation and Analysis of Essential Oils of Boswellia Oleogum Resins

Essential oils were obtained by hydrodistillation of *Boswellia* spp. oleogum resins. For each approach, respectively, 100 g oleogum resin of *B. sacra* (sample # 1), *B. serrata* (sample # 18), *B. carterii* (sample # 25), *B. frereana* (sample # 38), *B. dalzielli* (sample # 12), *B. papyrifera* (sample # 15), *B. neglecta* (sample # 35), or *B. rivae* (sample # 37) were added to 250 mL water and mixed for several minutes until a thick homogenous dispersion was formed. For hydrodistillation, the mixtures were subsequently heated for 6–7 h at 120–125 °C with continuous stirring. The obtained essential oils were carefully separated from the aqueous phase with the help of a separating funnel, yielding 1.2–9.7% essential oils (*w/w*), respectively.

GC-FID analysis of essential oils was performed on an Agilent 7890A system equipped with a DB-5 capillary column (10 m × 0.10 mm × 0.10 µm) and a DB-Wax capillary column (10 m × 0.10 mm × 0.10 µm). Hydrogen was used as carrier gas (0.7 mL/min, constant flow). Oven temperature started at 35 °C for 1 min, followed by linear gradient to 260 °C by 9 °C/min, and then held for 10 min. Essential oil samples were diluted at roughly 20 mg/mL in GC-grade pentane and were injected with a volume of 1 µL and a split of 50:1 with an inlet temperature of 250 °C.

GC-MS analysis of essential oils was performed on an Agilent 7890B/5977B system equipped with a HP-5MS capillary column (30 m × 0.25 mm × 0.25 µm). Helium was used as a carrier gas (1.0 mL/min, constant flow). Oven temperature started at 40 °C for 2 min, followed by linear gradient to 270 °C by 4 °C/min, and then held for 5 min. Essential oil samples were injected neat with a volume of 0.5 µL and a split of 50:1, with an inlet temperature of 250 °C. Compounds were identified using their retention indexes as calculated from a homologous series of *n*-alkanes and mass spectra comparisons with NIST14 libraries, in-house libraries, and specialist literature [61,62].

### 3.6. Analysis of Antiproliferative and Cytotoxic Effects In Vitro

Triple-negative human breast cancer cells MDA-MB-231 (ATCC, Rockville, MD, USA), CAL-51 (DSMZ, Braunschweig, Germany), and CAL-148 (DSMZ), as well as peripheral blood mononuclear cells (PBMC) were analyzed. PBMC were isolated from whole venous blood from healthy male donors via density gradient centrifugation using Biocoll (Biochrom GmbH, Berlin, Germany). Informed consent of the volunteers and approval of the institutional Ethics Committee (#177/18) were obtained. MDA-MB-231 cells were cultured in Dulbecco’s Modified Eagle Medium (4.5 g/L glucose, GlutaMax; Life Technologies, Carlsbad, CA, USA) supplemented with 10% FCS (fetal calf serum), 0.1 mM MEM (minimum essential medium) non-essential amino acids, 100 U/mL penicillin, and 100 mg/mL streptomycin. CAL-51 cells were cultured in Dulbecco´s Modified Eagle Medium (4.5 g/L glucose; Life Technologies, Carlsbad CA, USA) supplemented with 10% FCS, 100 U/mL penicillin, and 100 mg/mL streptomycin. CAL-148 cells were cultured in Dulbecco´s Modified Eagle Medium (4.5 g/L glucose; Life Technologies) supplemented with 10% FCS, 100 U/mL penicillin, 100 mg/mL streptomycin, and 10 ng/mL epidermal growth factor (EGF). All cancer cell lines were cultured in a humidified incubator at 37 °C and 5% CO_2_ (MDA-MB-251 und CAL-51) or 10% CO_2_ (CAL-148) atmosphere. Cells were sub-cultured according to the supplier’s recommendations after reaching 80% confluence. For the experiments, 5000 cells/well of the respective cancer cell lines or 300,000 PBMC/well were seeded into 96-well plates in medium supplemented with 10% FCS overnight and treated 24 h after seeding. Respective concentrations of the samples were applied by means of a Tecan D300e Digital Dispenser (Tecan, Männedorf, Switzerland). After a 72 h incubation period, cell viability was analyzed by addition of 2,3-bis-(2-methoxy-4-nitro-5-sulfophenyl)-2H-tetrazolium-5-carboxanilide salt (XTT; AppliChem GmbH, Darmstadt, Germany). Absorbance of the formed orange formazan dye was measured using an Infinite M1000 PRO Tecan plate reader (Tecan) at λ = 450 nm with a λ = 630 nm reference filter. For quantification of cell viability, the blank values containing the respective compounds in the according concentration were subtracted and the percentage of viable cells was calculated by normalization to the vehicle control.

### 3.7. Human Tumors Xenografted onto the Chick Choriallantoic Membrane 

For investigation of antitumor activity against TNBC xenografts in vivo, 0.7 × 10^6^ MDA-MB-231 cells in medium/matrigel (1/1, *v/v*) were xenografted onto the chick chorioallantoic membrane (CAM) of fertilized chick eggs 7 days after fertilization. For the next three consecutive days, cells were treated topically with 10 or 50 µM of α-KBA and α-AKBA respectively, or 10 µM doxorubicin dissolved in 0.9% NaCl (vehicle control: 0.5% DMSO). Tumors were collected, imaged, fixed, and embedded in paraffin for analysis by immunohistochemistry on the fourth day after treatment initiation. Tumor volumes (mm^3^) were calculated with the formula length (mm) × width^2^ (mm^2^) × π/6 [63]. For immunohistochemical analysis, slices (D = 5 µm) of the collected tumor specimens were stained using hematoxylin and eosin. Furthermore, the slices were stained with antibodies against the nuclear proliferation marker Ki-67 (M7240; Dako, Glostrup, Denmark). DNA strand breaks were stained by deoxynucleotidyl transferase dUTP (deoxyuridine triphosphate) nick end labeling (TUNEL; Roche, Basel, Switzerland) according to the manufacturer´s recommendations to visualize apoptosis in vivo. Images were recorded by means of an Axio Lab.A1 microscope (Carl Zeiss, Oberkochen, Germany) and a Zeiss 2/3” CMOS camera using Progres Gryphax software (Jenoptik, Jena, Germany).

### 3.8. Statistical Analysis

Experiments for HPLC-MS/MS quantifications were carried out in duplicates and all in vitro and in vivo experiments in at least three independent experiments. All in vitro experiments were additionally performed in triplicates. The data are expressed as mean ± standard deviation (SD) or standard error of the mean (SEM), as indicated. Statistical analysis was performed with Minitab 18 software (Minitab, Munich, Germany), SigmaPlot 14.0 software (Systat Software Inc., San Jose, CA, USA), and Valoo 2.10 software (Applica, Bremen, Germany). All data were tested for normal distribution by the Anderson-Darling test and equality of variances by Levene´s test. Sample groups were compared by one-way analysis of variance (ANOVA) and post hoc by Dunett´s test or by Fisher’s least significant difference (LSD) test. Comparison of two sample groups was executed by Student´s *t*-test. Results with 0.001 ≤ *p* < 0.05 were considered as statistically significant and *p* < 0.001 as highly significant. Principal component analysis (PCA) was derived from a correlation matrix of the raw data yielding

(3) PC1=1.415 [α-KBA]+1.437 [β-KBA]+1.636 [α-AKBA]+1.502 [β-AKBA]

and

(4) PC2=−1.709 [α-KBA]−1.345 [β-KBA]+1.001 [α-AKBA]+1.807 [β-AKBA]

with concentrations of the corresponding KBAs or AKBAs in µg/mg oleogum resin given in square brackets.

## 4. Conclusions

In oleogum resins of *Boswellia* spp., a novel triterpenoid, 11-keto-α-boswellic acid (α-KBA), was identified. α-KBA was synthesized and characterized by 1D and 2D NMR and HR-MS and a highly sensitive and accurate HPLC-MS/MS method for selective evidence and quantification of individual keto-boswellic acids was developed. By means of this method, 41 *Boswellia* oleogum resins were investigated and it could be demonstrated that in addition to the well-known 11-keto-β-boswellic acid (β-KBA), its α-constitutional isomer, α-KBA, exists in *Boswellia* spp. On the basis of the individual keto-boswellic acid contents and with inclusion of the chemical composition of essential oils and the composition of the oleogum resins’ boswellic acids, a chemotaxonomic characterization of different *Boswellia* species could be achieved. Moreover, α-KBA exhibited cytotoxicity against human treatment-resistant, triple-negative breast cancer (TNBC) cell lines in vitro and in vivo. However, the β-isomer, β-KBA, and its acetylation enhanced the lipophilicity as well as the cytotoxic efficacy. These results provide a deeper insight into the structure–activity relationship of boswellic acids and point to the necessity for further studies aiming at the development of new anticancer drugs.

## Figures and Tables

**Table 1:** ^1^H and ^13^C NMR (proton and carbon-13 nuclear magnetic resonance) assignments for 11-keto-α-boswellic acid (α-KBA) in DMSO-*d_6_* (deuterated dimethyl sulfoxide). Chemical shifts δ_H_/δ_C_ in ppm (multiplicity, ^3^J_HH_ in Hz, topicity).

Position, C	δ_C_	δ_H_
1	33.6	1.28 (ddd, 14/14/4, α)
		2.27 (bd, 14, β)
2	25.9	1.33 (ddd, 4/4/14, α)
		2.05 (dd, 14/14, β)
3	68.7	3.77 (bs, β)
4	46.7	-
5	47.6	1.39 (m, α)
6	18.6	1.61 (m, β)
		1.76 (dd, 14/4, α)
7	32.3	1.36 (m, β)
		1.60 (m, α)
8	44.9	-
9	60.1	2.35 (s, α)
10	37.1	-
11	198.9	-
12	127.3	5.47 (s)
13	170.3	-
14	43.1	-
15	25.9	1.74 (dd, 14/4, α)
		2.06 (dd, 14/4, β)
16	25.8	0.91 (bd, 14, β)
		1.15 (bd, 14, α)
17	32.0	-
18	46.9	2.12 (dd, 14/4, β)
19	44.7	1.00 (bd, 14, β)
		1.70 (dd, 14/14, α)
20	30.8	-
21	33.9	1.11 (m, β)
		1.39 (m, α)
22	36.0	1.25 (m, β)
		1.40 (m, α)
23	24.4	1.13 (s, α)
24	178.7	-
25	13.1	0.99 (s, β)
26	18.2	1.05 (s, β)
27	23.1	1.35 (s, α)
28	28.4	0.83 (s, β)
29	23.4	0.88 (s, β)
30	32.8	0.89 (s, α)

**Table 2:** Experimental design with level conditions for three independent variables A, B, and C (coded and uncoded) and the chromatographic resolutions, *R*, for separation of α-KBA and β-KBA as well as α-AKBA/β-AKBA as dependent variables. The level conditions of experiment 5 enabled a successful separation of all constitutional isomers with required *R* ≥ 1.5. The conditions of experiment 11 were not appropriate to elute AKBA isomers from the column.

Exp.	Independent Variables (Coded)			Independent Variables (Uncoded)			Dependent Variables Resolution *R*	
	A	B	C	A [%]	B [%/min]	C [mL/min]	α-KBA/β-KBA	α-AKBA/β-AKBA
1	–1	–1	–1	8.11	1.22	0.51	1.40	3.03
2	1	–1	–1	31.89	1.22	0.51	0.84	2.69
3	–1	1	–1	8.11	4.78	0.51	0.84	1.89
4	1	1	–1	31.89	4.78	0.51	0.51	1.42
5	–1	–1	1	8.11	1.22	0.69	1.52	3.04
6	1	–1	1	31.89	1.22	0.69	0.80	2.65
7	–1	1	1	8.11	4.78	0.69	0.97	2.00
8	1	1	1	31.89	4.78	0.69	0.62	1.95
9	–α	0	0	0.00	3.00	0.60	1.18	2.14
10	α	0	0	40.00	3.00	0.60	0.64	1.89
11	0	–α	0	20.00	0.00	0.60	1.50	N/A
12	0	α	0	20.00	6.00	0.60	0.63	1.73
13	0	0	–α	20.00	3.00	0.45	0.92	1.95
14	0	0	α	20.00	3.00	0.75	1.16	2.12
15	0	0	0	20.00	3.00	0.60	0.77	2.05

**Table 3:** Contents of 11-keto-α-boswellic acid (α-KBA), 11-keto-β-boswellic acid (β-KBA), acetyl-11-keto-α-boswellic acid (α-AKBA), and 11-keto-β-boswellic acid (β-AKBA) in *Boswellia* oleogum resins. Quantification by high-performance liquid chromatography with tandem mass spectrometry (HPLC-MS/MS) in duplicates. Average values of respective species are expressed as mean ± standard deviation (SD); contents below the limit of quantification as <LOQ.

Sample			11-Keto-Boswellic Acids (KBAs)				Acetyl-11-Keto-Boswellic Acids (AKBAs)			
#	Species	Origin	Ratio [%]		Content [µg/mg]		Ratio [%]		Content [µg/mg]	
			β-KBA	α-KBA	β-KBA	α-KBA	β-AKBA	α-AKBA	β-AKBA	α-AKBA
1	*B. sacra*	Oman	96.4	3.6	2.618	0.098	98.3	1.7	41.088	0.731
2	*B. sacra*	Oman	93.8	6.2	2.658	0.175	98.2	1.8	28.646	0.532
3	*B. sacra*	Oman	94.4	5.6	1.153	0.068	98.8	1.2	33.956	0.416
4	*B. sacra*	Oman	98.2	1.8	1.081	0.020	99.2	0.8	32.642	0.267
5	*B. sacra*	Oman	98.9	1.1	2.138	0.024	99.5	0.5	35.694	0.197
	*B. sacra*	Oman	96.3	3.7	2.209	0.085	98.6	1.4	21.465	0.308
7	*B. sacra*	Oman	97.2	2.8	1.571	0.046	99.1	0.9	37.143	0.326
8	*B. sacra*	Oman	97.2	2.8	2.041	0.059	99.0	1.0	32.994	0.350
9	*B. sacra*	Oman	93.4	6.6	0.930	0.066	98.2	1.8	25.617	0.466
10	*B. sacra*	Oman	94.6	5.4	1.028	0.059	98.4	1.6	21.416	0.359
11	*B. sacra*	Oman	94.0	6.0	1.084	0.069	98.3	1.7	29.549	0.509
**Mean**			**95.8**	**4.2**	**1.683**	**0.070**	**98.7**	**1.3**	**30.928**	**0.406**
*SD*			*1.8*	*1.8*	*0.636*	*0.040*	*0.4*	*0.4*	*6.002*	*0.142*
12	*B. dalzielli*	Burkina Faso	93.4	6.6	10.893	0.770	96.1	3.9	51.329	2.096
13	*B. dalzielli*	Nigeria	96.2	3.8	9.482	0.377	97.7	2.3	70.521	1.677
14	*B. dalzielli*	Senegal	92.6	7.4	12.840	1.019	96.0	4.0	65.623	2.709
**Mean**			**94.1**	**5.9**	**11.072**	**0.722**	**96.6**	**3.4**	**62.491**	**2.161**
*SD*			*1.9*	*1.9*	*1.686*	*0.324*	*0.9*	*0.9*	*9.972*	*0.519*
15	*B. papyrifera*	Ethiopia	97.2	2.8	4.261	0.123	93.6	6.4	41.109	2.833
16	*B. papyrifera*	Eritrea	92.2	7.8	3.142	0.264	96.3	3.7	26.813	1.031
17	*B. papyrifera*	Sudan	94.5	5.5	3.013	0.174	96.6	3.4	16.235	0.574
**Mean**			**94.7**	**5.3**	**3.472**	**0.187**	**95.5**	**4.5**	**28.052**	**1.479**
*SD*			*2.5*	*2.5*	*0.686*	*0.071*	*1.7*	*1.7*	*12.483*	*1.194*
18	*B. serrata*	India	98.8	1.2	5.266	0.064	95.6	4.4	10.823	0.501
19	*B. serrata*	India	98.9	1.1	22.045	0.244	98.1	1.9	18.338	0.354
20	*B. serrata*	India	98.5	1.5	3.043	0.047	97.0	3.0	13.233	0.411
21	*B. serrata*	India	98.5	1.5	5.705	0.088	95.1	4.9	8.141	0.420
22	*B. serrata*	India	95.4	4.6	9.346	0.451	96.3	3.7	12.766	0.491
23	*B. serrata*	India	94.1	5.9	5.885	0.371	95.3	4.7	8.816	0.437
24	*B. serrata*	India	96.0	4.0	3.886	0.161	95.7	4.3	7.720	0.348
**Mean**			**97.2**	**2.8**	**7.882**	**0.204**	**96.1**	**3.9**	**11.405**	**0.423**
*SD*			*2.0*	*2.0*	*6.554*	*0.158*	*1.1*	*1.1*	*3.753*	*0.060*
25	*B. carterii*	Somalia	79.4	20.6	0.063	0.016	85.2	14.8	0.088	0.015
26	*B. carterii*	Somalia	79.7	20.3	0.013	0.003	88.2	11.8	0.053	0.007
27	*B. carterii*	Somalia	74.9	25.1	0.107	0.036	N/A	N/A	0.001	<LOQ
28	*B. carterii*	Somalia	74.5	25.5	0.644	0.221	90.3	9.7	0.005	0.001
29	*B. carterii*	Somalia	94.9	5.1	6.123	0.331	98.7	1.3	48.724	0.619
**Mean**			**80.7**	**19.3**	**1.390**	**0.121**	**90.6**	**9.4**	**9.774**	**0.128**
*SD*			*8.3*	*8.3*	*2.658*	*0.147*	*5.8*	*5.8*	*21.774*	*0.274*
30	*B. neglecta*	Somalia	67.1	32.9	0.968	0.476	84.1	15.9	0.037	0.007
31	*B. neglecta*	Somalia	72.1	27.9	1.128	0.436	83.4	16.6	0.119	0.024
32	*B. neglecta*	Somalia	67.5	32.5	1.168	0.563	81.6	18.4	0.039	0.009
33	*B. neglecta*	Somalia	81.5	18.5	0.517	0.118	81.9	18.1	0.066	0.015
34	*B. neglecta*	Somalia	73.6	26.4	0.177	0.063	81.9	18.1	0.020	0.004
35	*B. neglecta*	Kenya	72.8	27.2	0.045	0.017	90.3	9.7	0.016	0.002
36	*B. neglecta*	Kenya	N/A	N/A	<LOQ	<LOQ	N/A	N/A	0.002	<LOQ
**Mean**			**72.4**	**27.6**	**0.572**	**0.239**	**83.9**	**16.1**	**0.043**	**0.009**
*SD*			*5.2*	*5.2*	*0.514*	*0.242*	*3.3*	*3.3*	*0.039*	*0.008*
37	*B. rivae*	Ethiopia	**42.3**	**57.7**	**0.016**	**0.022**	**92.5**	**7.5**	**0.051**	**0.004**
38	*B. frereana*	Somalia	N/A	N/A	<LOQ	<LOQ	N/A	N/A	<LOQ	<LOQ
39	*B. frereana*	Somalia	N/A	N/A	<LOQ	<LOQ	N/A	N/A	<LOQ	<LOQ
40	*B. frereana*	Somalia	N/A	N/A	<LOQ	<LOQ	N/A	N/A	<LOQ	<LOQ
**Mean**			**N/A**	**N/A**	**<LOQ**	**<LOQ**	**N/A**	**N/A**	**<LOQ**	**<LOQ**
*SD*			*-*	*-*	*-*	*-*	*-*	*-*	*-*	*-*
41	*B. occulta*	Somalia	**94.4**	**5.6**	**2.527**	**0.150**	**98.4**	**1.6**	**33.104**	**0.540**

**Table 4:** Chemical composition of *Boswellia* spp. oleogum resin essential oils. Relative quantification by gas chromatography and flame ionization detection (GC-FID) using internal normalization, areas in uncorrected %. List of main components (area >1%), plus the potential psychoactive diterpenoid incensole.

Compound	*B. sacra*	*B. serrata*	*B. carterii*	*B. frereana*	*B. dalzielli*	*B. papyrifera*	*B. neglecta*	*B. rivae*
Hashishene	0.32	5.24	0.12	2.89	1.01	0.03	0.01	0.12
α-Thujene	0.40	14.46	3.69	37.07	5.36	0.41	7.86	1.55
α-Pinene	71.44	14.71	52.16	24.41	70.29	1.58	46.95	42.33
Camphene	1.76	0.19	0.80	0.56	1.10	0.10	0.98	1.03
Thuja-2,4(10)-diene	0.75	0.06	0.26	0.36	1.08	0.03	0.47	0.58
Sabinene	2.39	2.34	2.77	4.87	0.68	0.26	0.22	0.99
β-Pinene	1.47	1.57	1.86	1.16	1.74	0.14	1.42	7.64
Myrcene	2.10	41.36	4.93	0.97	1.91	0.37	0.81	0.13
α-Phellandrene	0.30	0.40	3.16	0.11	0.03	0.02	0.11	0.16
Δ^3^-Carene	1.84	0.47	0.81	0.02	0.03	0.03	1.23	6.51
*para*-Cymene	0.82	1.10	4.50	8.20	1.54	0.29	4.61	7.86
Limonene	3.17	4.63	15.90	0.85	2.19	2.03	2.23	4.39
(*E*)-β-Ocimene	0.16	0.05	0.06	0.01	0.02	1.62	0.02	0.03
Linalool	0.04	0.66	0.02	0.26	0.17	1.00	0.08	0.07
*trans*-Pinocarveol	0.94	0.08	0.21	0.33	1.45	0.00	0.39	1.83
*trans*-Verbenol	0.79	0.09	0.37	0.37	1.12	0.02	0.45	2.52
α-Phellandren-8-ol	1.08	0.04	0.24	0.48	1.39	0.01	0.63	0.61
Terpinen-4-ol	0.28	0.09	0.19	1.73	0.19	0.07	16.81	0.69
α-Terpineol	0.03	0.09	0.06	0.26	0.11	0.00	4.00	0.38
Verbenone	0.64	0.03	0.16	0.31	0.74	0.00	0.66	1.76
Incensole	0.00	0.00	0.00	0.00	0.01	0.05	0.00	0.02
Methylchavicol	0.00	5.96	0.00	0.00	0.00	0.00	0.00	0.00
Octanol	0.00	0.00	0.00	0.00	0.00	6.96	0.01	0.00
Octyl acetate	0.00	0.00	0.00	0.00	0.00	77.67	0.00	0.00

**Table 5:** Cytotoxic efficacies of individual 11-keto-boswellic acid (KBA) and acetyl-11-keto-boswellic acids (AKBA) isomers against MDA-MB-231 cells in vitro. XTT assay, 72 h, *n* = 3 (all biological replicates were performed in triplicates). ALogP values calculated with BIOVIA Draw Ver. 20.1 software (Dassault Systemes, Vélizy-Villacoublay, France). Half maximal inhibitory concentration (IC_50_) values of β-KBA and β-AKBA from a previously published study [27]. Data are expressed as mean ± standard error of the mean (SEM).

Compound	IC_50_ [µM]		Lipophilicity,
	Mean	SEM	ALogP
α-KBA	41.96	4.63	5.66
β-KBA	25.45	0.87	5.70
α-AKBA	27.50	2.11	6.04
β-AKBA	6.55	0.21	6.08

## Data Availability

Data are contained within the article or supplementary material.

## Supplementary Materials

Figure S1: High-resolution mass spectrometry (HR-MS) of α-KBA, Figure S2: Tandem mass spectrometry (MS/MS) of α-KBA, Figure S3: ^1^H and ^13^C NMR spectra of α-KBA, Figure S4: ^1^H, ^1^H SELTOCSY (selective total correlation spectroscopy) spectra of α-KBA, Figure S5: ^1^H, ^1^H COSY (correlation spectroscopy) spectrum of α-KBA, Figure S6: ^1^H, ^1^H ROESY (rotating frame Overhauser enhancement spectroscopy) spectrum of α-KBA, Figure S7: ^1^H, ^13^C HSQC (heteronuclear single quantum coherence spectroscopy) spectrum of α-KBA, and Figure S8: ^1^H, ^13^C HMBC (heteronuclear multiple bond correlation spectroscopy) spectrum of α-KBA.

## Sample Availability

Samples of the compounds and *Boswellia* oleogum resins are available from the authors.

## Appendix A

**Table A1:** HPLC-MS/MS validation data: Regression equation, limit of detection (LOD), and limit of quantification (LOQ).

Compound	Regression Equation ^1^				LOD ^2^ [ng/mL]	LOQ ^2^ [ng/mL]
	$y_{i}=a\times c_{i}+b$					
	Slope (a)	Offset (b)	R^2^	Lin. Test		
α-KBA	164.7	1130.8	0.9996	linear	6.0	21.1
β-KBA	170.9	366.8	0.9999	linear	4.8	17.0
α-AKBA	408.6	1000.1	0.9997	linear	2.3	8.5
β-AKBA	420.2	1002.5	0.9997	linear	3.7	13.4

^1^ Regression equations of 10-point calibration (1–1000 ng/mL); y_i_: peak area of the corresponding compound [cps]; c_i_: concentration of the corresponding compound [ng/mL]; linearity (lin.) test with 1% level of significance. ^2^ LOD and LOQ according to DIN 32645 on the basis of a 7-point calibration (1–100 ng/mL).

**Table A2:** HPLC-MS/MS validation data: Precision and recovery.

Compound	Intraday and Interday Precision ^1^ (RSD)				Recovery ^2^ [%]	
	Low Level [%]		High Level [%]			
	Intraday	Interday	Intraday	Interday	Mean	SD
α-KBA	5.1	1.6	5.1	6.3	97.2	4.1
β-KBA	6.0	2.7	5.4	3.5	98.9	4.1
α-AKBA	5.2	7.5	2.6	7.6	90.9	4.6
β-AKBA	4.3	4.8	3.1	8.7	98.4	3.2

^1^ RSD: relative standard deviation [%]; low level: 50 ng/mL; high level: 500 ng/mL; intraday repetitions: *n* = 5; interday repetitions: *n* = 4. ^2^ Method of standard addition; sample # 25 (500 µg/mL) spiked on three levels (125, 250, and 500 ng/mL).
