# Supplementary material for: 11-Keto-α-Boswellic Acid, a Novel Triterpenoid from Boswellia spp. with Chemotaxonomic Potential and Antitumor Activity against Triple-Negative Breast Cancer Cells
Source: Molecules. 2021 Jan 12;26(2):366. doi: 10.3390/molecules26020366 (PMC7828217; doi:10.3390/molecules26020366)
Supplement: Supplementary file 1 [file molecules-26-00366-s001.pdf]

Supplementary Materials

11-Keto-α-boswellic Acid, a Novel Triterpenoid from *Boswellia* spp. with Chemotaxonomic Potential and Antitumor Activity against Triple-Negative Breast Cancer Cells

Michael Schmiech [1], Judith Ulrich [1], Sophia Johanna Lang [1], Christian Paetz [2], Alexis St-Gelais [3], Berthold Büchele [1], Tatiana Syrovets [1,*,†], and Thomas Simmet [1,*,†]

[1] Institute of Pharmacology of Natural Products and Clinical Pharmacology, Ulm University, 89081 Ulm, Germany; michael.schmiech@uni-ulm.de (M.S.); judith.ulrich@uni-ulm.de (J.U.); sophia.lang@uni-ulm.de (S.J.L.); berthold.buechele@t-online.de (B.B.)

[2] Max Planck Institute for Chemical Ecology, 07745 Jena, Germany; cpaetz@ice.mpg.de (C.P.)

[3] Laboratoire PhytoChemia, Québec G7J 1H4, Canada; a.st-gelais@phytochemia.com (A.S.)

* Correspondence: tatiana.syrovets@uni-ulm.de (T.S.); thomas.simmet@uni-ulm.de (Th.S.); Tel.: +49-731-500-65604 (T.S.); +49-731-500-65600 (Th.S.)

† These authors contributed equally to this work

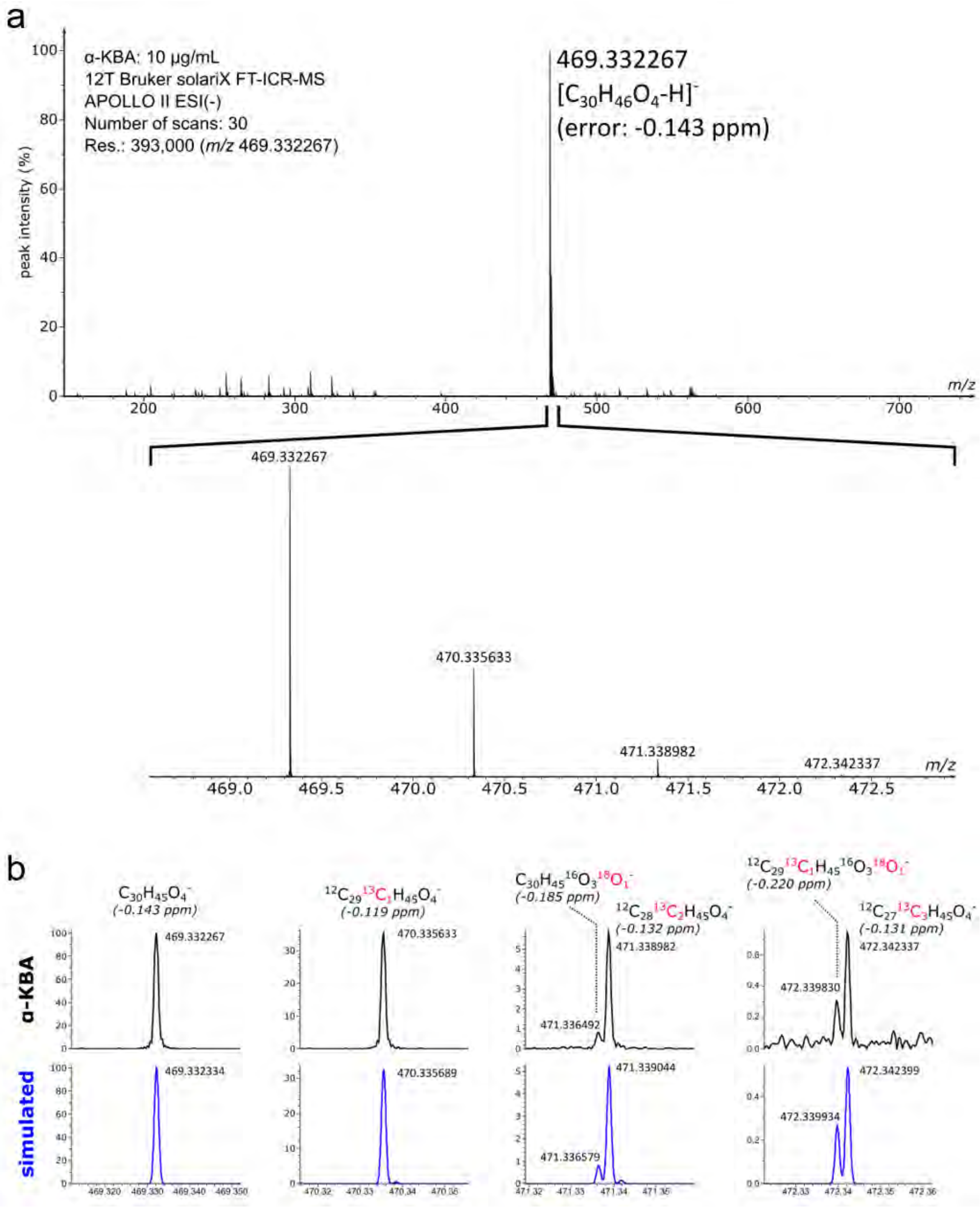

Figure S1. High-resolution mass spectrometry (HR-MS) of *α*-KBA. (**a**) HR mass spectrum with an exact mass at *m*/*z* 469.332267 for [M-H]$^-$ (calcd.: 469.332333, error: -0.143 ppm). (**b**) The isotope pattern and the individual exact masses corresponded to predicted data.

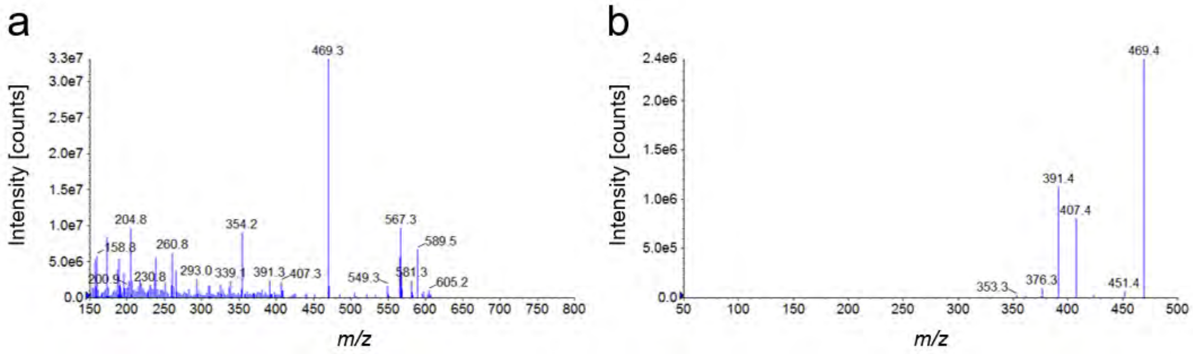

Figure S2. Tandem mass spectrometry (MS/MS) of α-KBA. (**a**) Mass spectrum with *m/z* 469.3 ([M-H]$^-$) as precursor ion. (**b**) Product ion mass spectrum with characteristic fragments at *m/z* 353.3, 376.3, 391.4, 407.4, and 451.4.

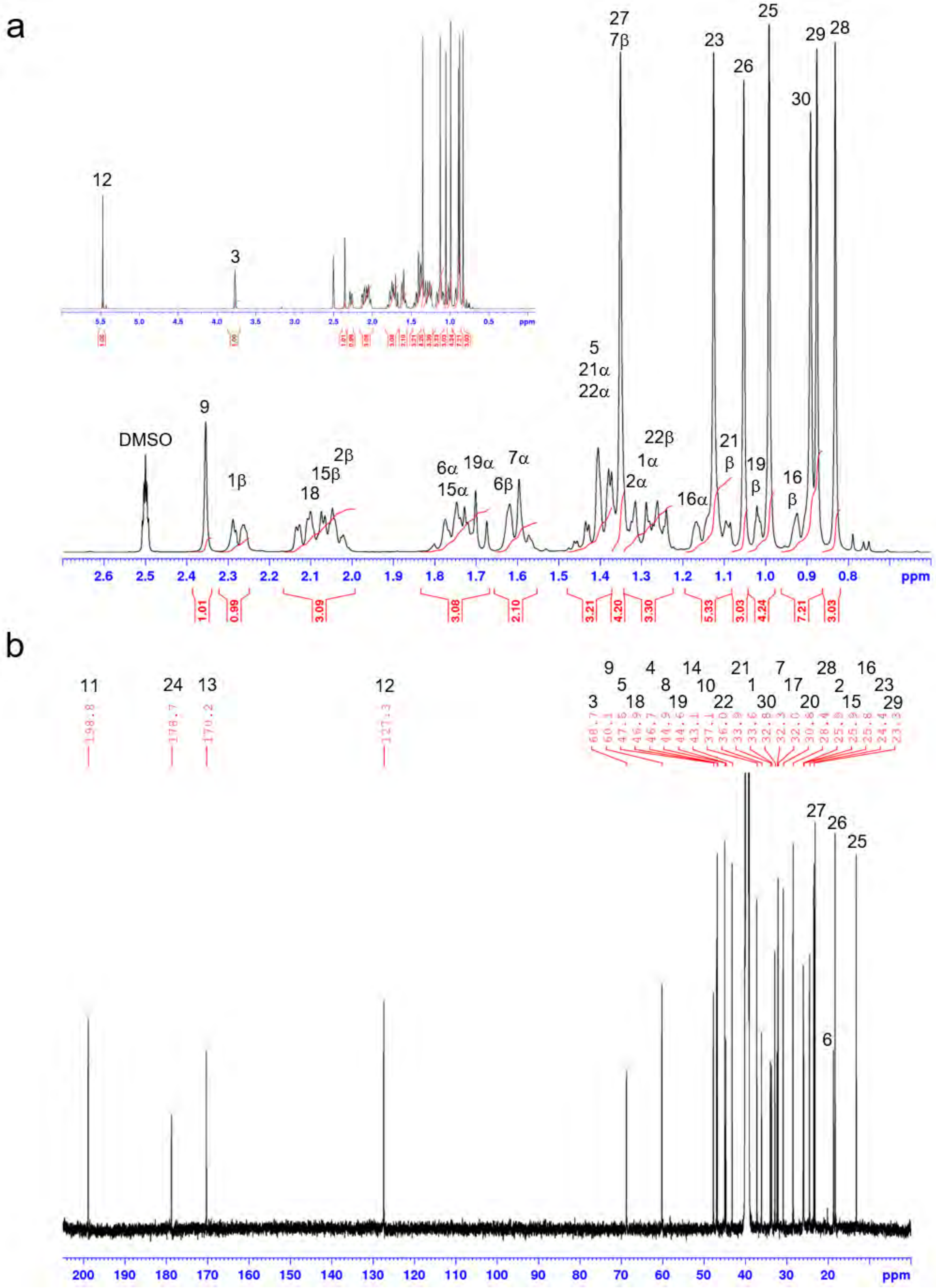

Figure S3. ^{1}H and ^{13}C NMR spectra of *α*-KBA (in DMSO-*d*$_6$). (**a**) ^{1}H NMR spectrum. (**b**) ^{13}C NMR spectrum. Assigment of signals according to Figure 3a and Table 1 of the main text.

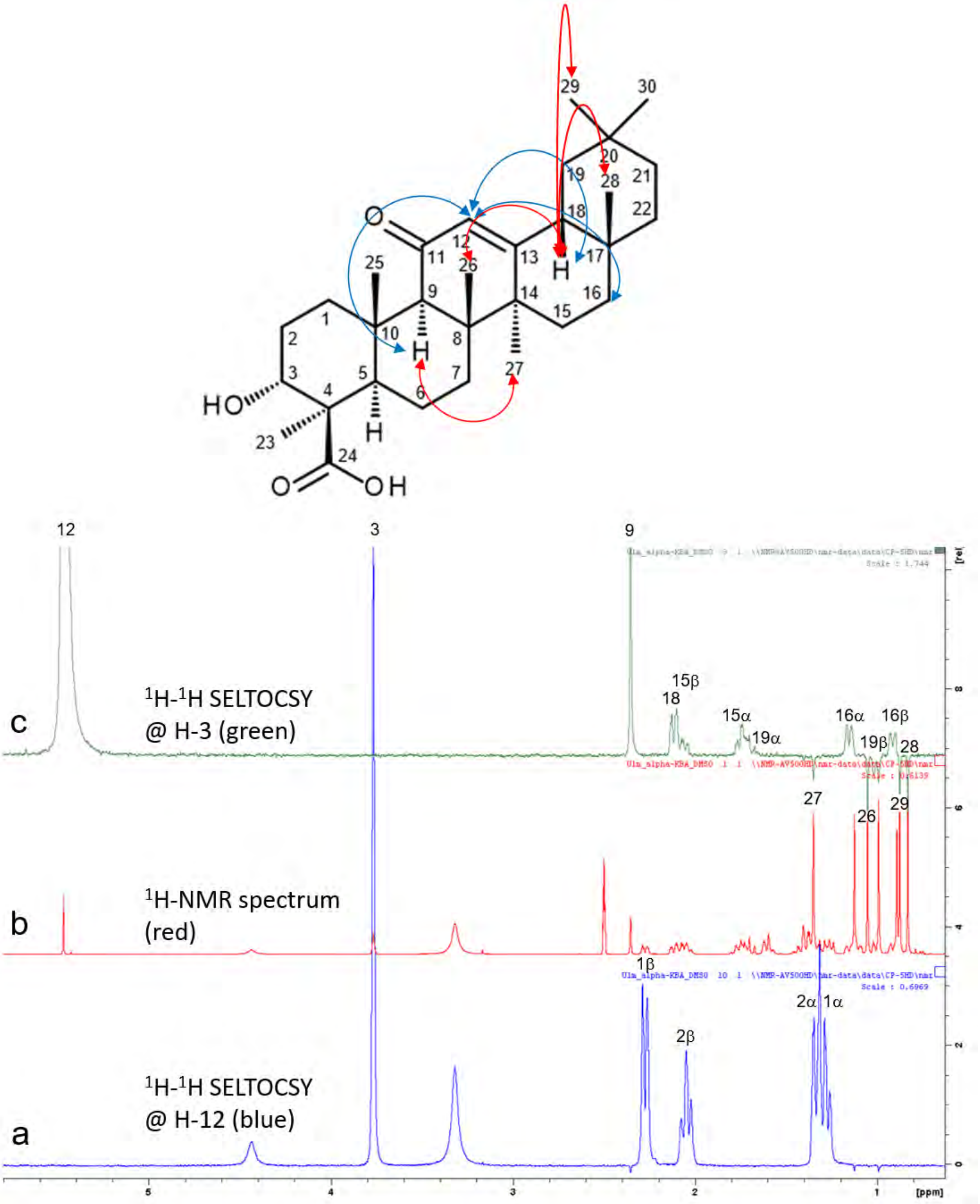

Figure S4. ^{1}H, ^{1}H SELTOCSY (selective total correlation spectroscopy) spectra of α-KBA (in DMSO-*d*$_6$). Comparison of the ^{1}H NMR spectrum (**b**) with ^{1}H, ^{1}H SELTOCSY spectra. (**a**) Transmitter frequency at δ_H 3.77 (H-3). (**c**) Transmitter frequency at δ_H 5.47 (H-12).

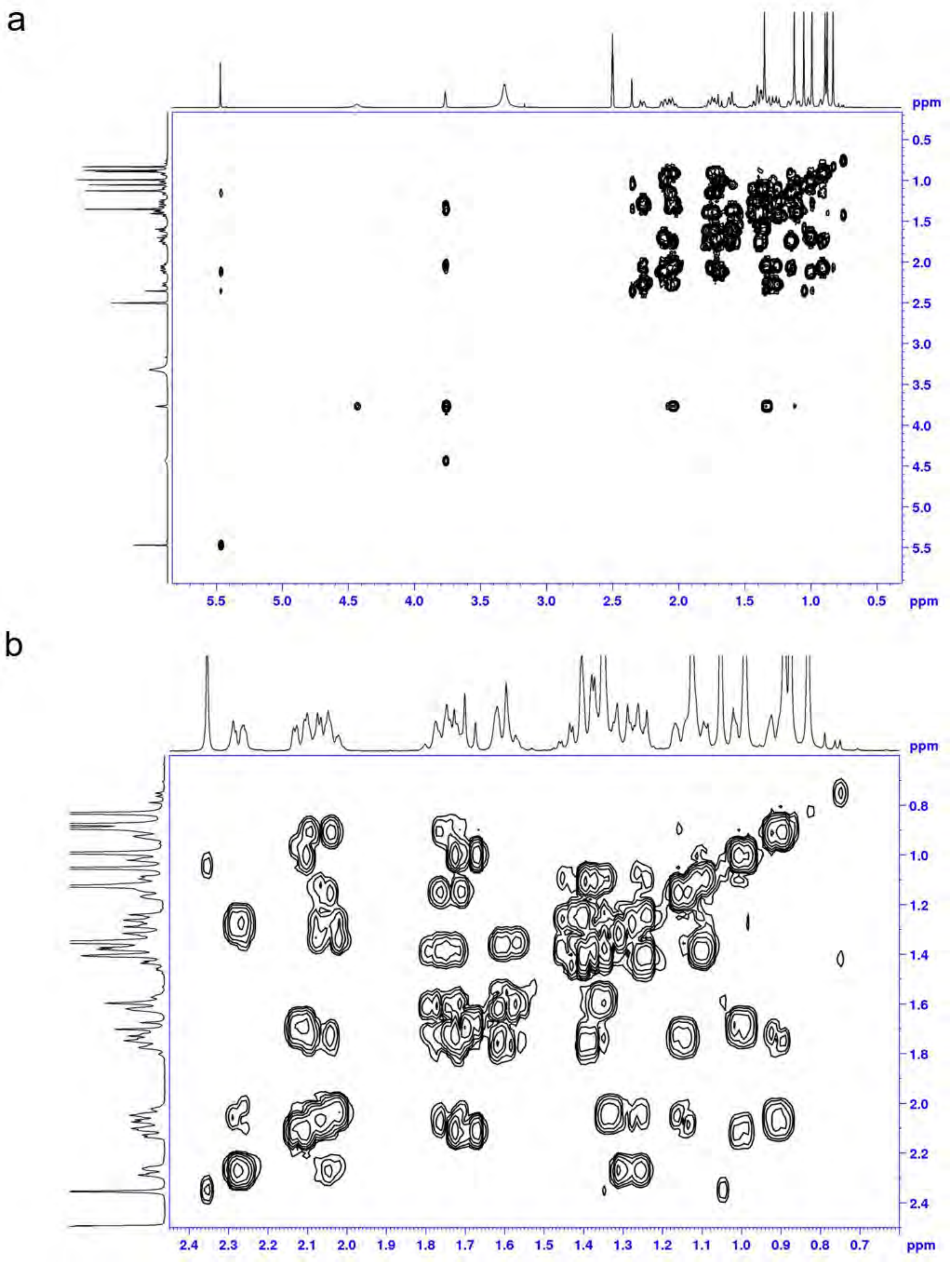

Figure S5. ^{1}H, ^{1}H COSY (correlation spectroscopy) spectrum of α-KBA (in DMSO-d_6). (**a**) Full spectrum. (**b**) Enlarged section.

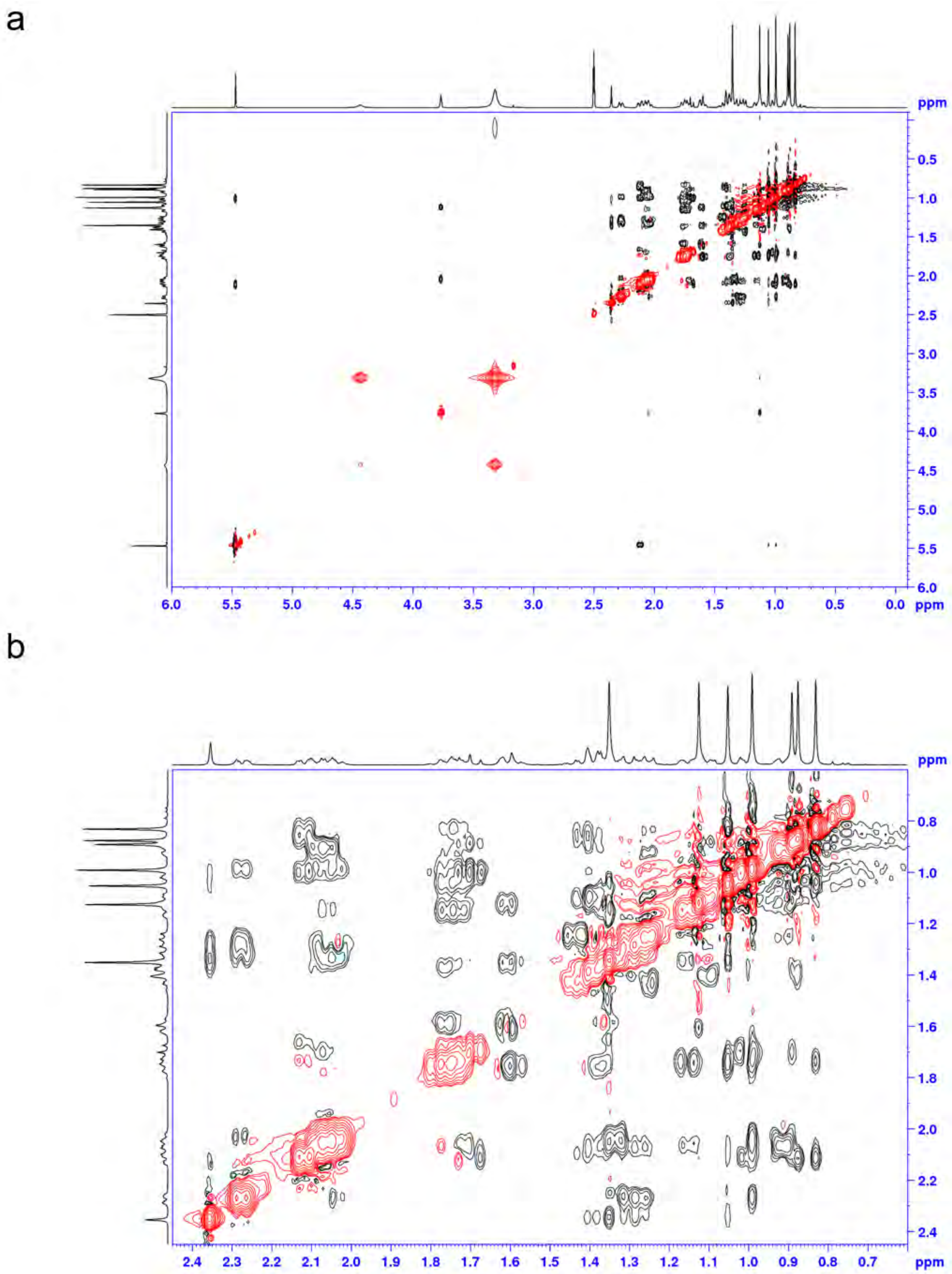

Figure S6. ^{1}H, ^{1}H ROESY (rotating frame Overhauser enhancement spectroscopy) spectrum of α-KBA (in DMSO-d_6). (**a**) Full spectrum. (**b**) Enlarged section.

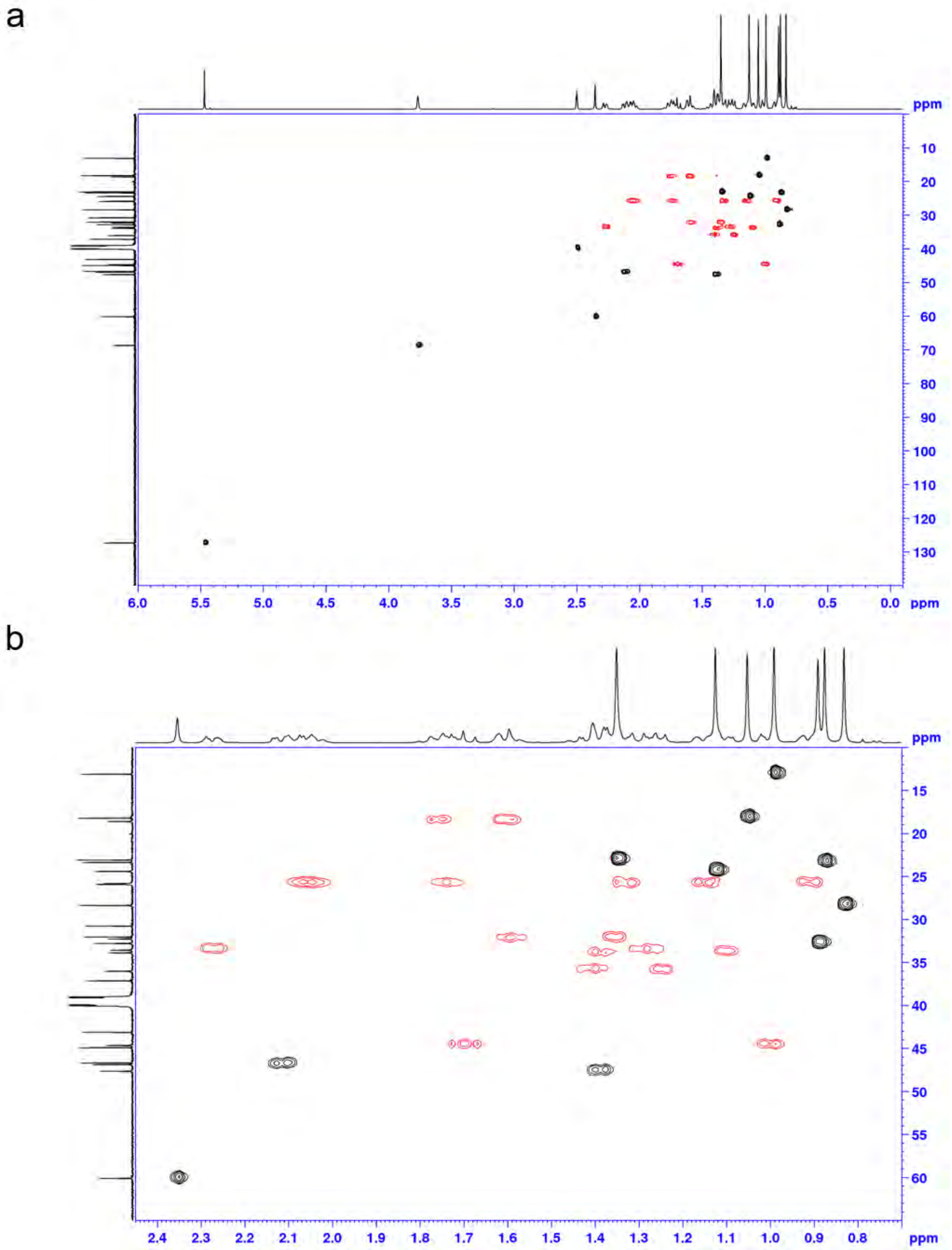

Figure S7. ^{1}H, ^{13}C HSQC (heteronuclear single quantum coherence spectroscopy) spectrum of α-KBA (in DMSO-d_6). (**a**) Full spectrum. (**b**) Enlarged section.

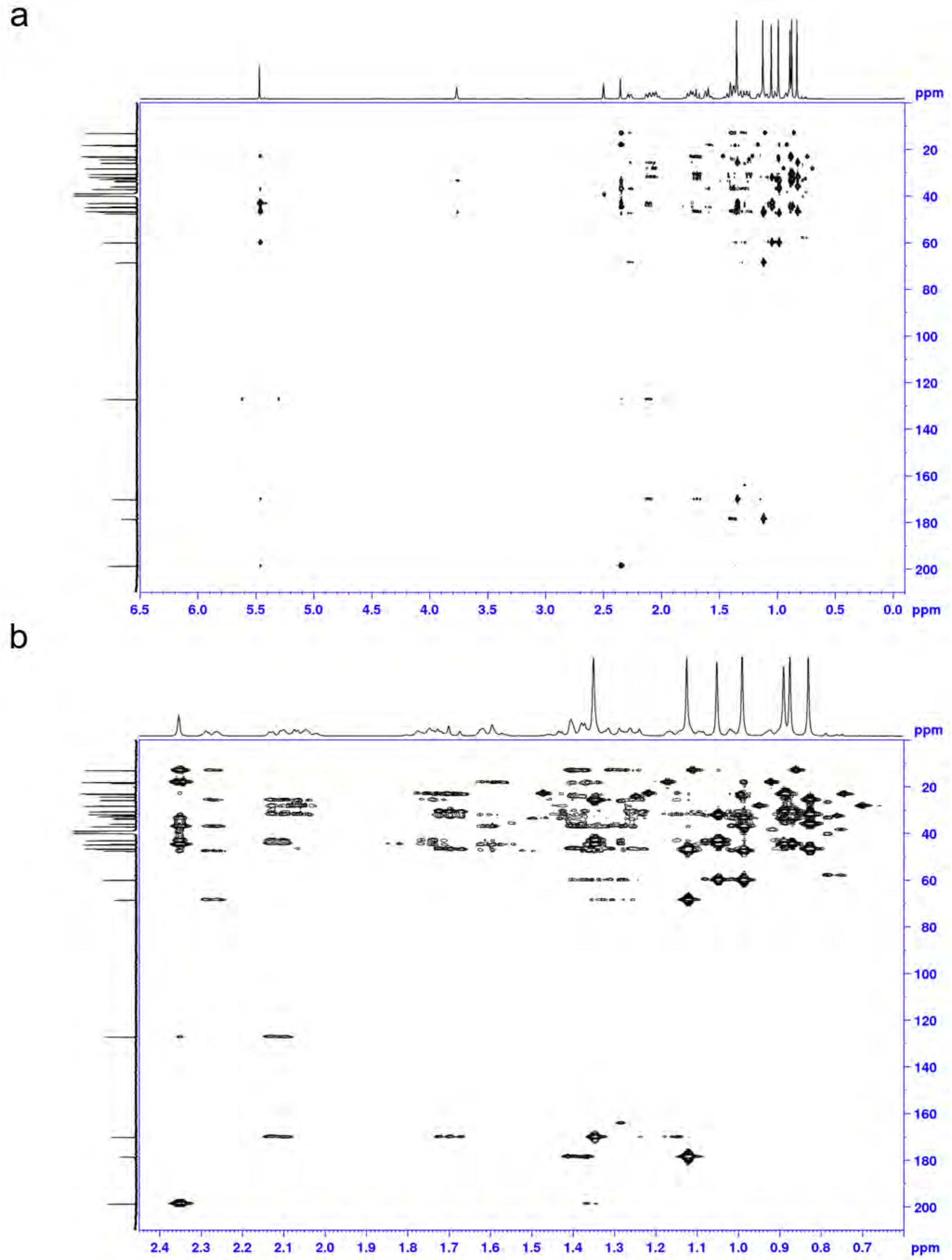

Figure S8. ^{1}H, ^{13}C HMBC (heteronuclear multiple bond correlation spectroscopy) spectrum of α-KBA (in DMSO-d_6). (**a**) Full spectrum. (**b**) Enlarged section.
